# Reassessing Domain Architecture Evolution of Metazoan Proteins: Major Impact of Errors Caused by Confusing Paralogs and Epaktologs

**DOI:** 10.3390/genes2030516

**Published:** 2011-08-02

**Authors:** Alinda Nagy, László Bányai, László Patthy

**Affiliations:** Institute of Enzymology, Biological Research Center, Hungarian Academy of Sciences, Budapest H-1113, Hungary; E-Mails: nagya@enzim.hu (A.N.); banyai@enzim.hu (L.B.)

**Keywords:** domain architecture, epaktologs, evolution of domain architecture, multidomain protein, Paralogs

## Abstract

In the accompanying paper (Nagy, Szláma, Szarka, Trexler, Bányai, Patthy, Reassessing Domain Architecture Evolution of Metazoan Proteins: Major Impact of Gene Prediction Errors) we showed that in the case of UniProtKB/TrEMBL, RefSeq, EnsEMBL and NCBI's GNOMON predicted protein sequences of Metazoan species the contribution of erroneous (incomplete, abnormal, mispredicted) sequences to domain architecture (DA) differences of orthologous proteins might be greater than those of true gene rearrangements. Based on these findings, we suggest that earlier genome-scale studies based on comparison of predicted (frequently mispredicted) protein sequences may have led to some erroneous conclusions about the evolution of novel domain architectures of multidomain proteins. In this manuscript we examine the impact of confusing paralogous and epaktologous multidomain proteins (*i.e.*, those that are related only through the independent acquisition of the same domain types) on conclusions drawn about DA evolution of multidomain proteins in Metazoa. To estimate the contribution of this type of error we have used as reference UniProtKB/Swiss-Prot sequences from protein families with well-characterized evolutionary histories. We have used two types of paralogy-group construction procedures and monitored the impact of various parameters on the separation of true paralogs from epaktologs on correctly annotated Swiss-Prot entries of multidomain proteins. Our studies have shown that, although public protein family databases are contaminated with epaktologs, analysis of the structure of sequence similarity networks of multidomain proteins provides an efficient means for the separation of epaktologs and paralogs. We have also demonstrated that contamination of protein families with epaktologs increases the apparent rate of DA change and introduces a bias in DA differences in as much as it increases the proportion of terminal over internal DA differences. We have shown that confusing paralogous and epaktologous multidomain proteins significantly increases the apparent rate of DA change in Metazoa and introduces a positional bias in favor of terminal over internal DA changes. Our findings caution that earlier studies based on analysis of datasets of protein families that were contaminated with epaktologs may have led to some erroneous conclusions about the evolution of novel domain architectures of multidomain proteins. A reassessment of the DA evolution of multidomain proteins is presented in an accompanying paper [1].

## 1. Introduction

Since formation of multidomain proteins with novel domain architectures (DA) is known to have played a major role in biological innovations of Metazoa [2,3] there is a growing interest in the genome-scale reconstruction of DA evolution with a view of defining the contribution of different genetic mechanisms. As pointed out in the accompanying paper [4], reliable reconstruction of the evolutionary history of DA of multidomain proteins requires that the protein sequences compared are valid, complete and correct and that the evolutionary relationship of the multidomain proteins compared is correctly defined.

As to the first requirement: we have found that in the case of UniProtKB/TrEMBL, RefSeq, EnsEMBL and NCBI's GNOMON predicted protein sequences of Metazoan species the contribution of sequence errors to domain architecture (DA) differences of orthologous proteins may be greater than those of true gene rearrangements, suggesting that sequence errors may have had a strong influence on the validity of the conclusions drawn from analyses of these databases.

As to the second requirement, we have shown that standard procedures used for orthology group construction are quite accurate, even for multidomain proteins.

In the present work we show that the standard procedures are much less reliable in defining groups of paralogs. This is due to the problem that, in the case of multidomain proteins, the major subtypes of homology (orthology, paralogy, pseudoparalogy) do not account for all types of relationships that may hold for two homologous multidomain proteins. Two homologous multidomain proteins of two different species are orthologous if they derive from the same protein/gene of the last common ancestor of the species, two homologous multidomain proteins are paralogous if they derive from the same gene that was duplicated within a genome and are pseudoparalogous if one of the genes was transferred by an interspecies transfer of genetic material [5,6]. These three, mutually exclusive subtypes of homology (*i.e.*, two homologous proteins can not be orthologous or paralogous or pseudoparalogous at the same time) do not account for all types of relationships that may exist between two homologous multidomain proteins.

**Figure 1:**
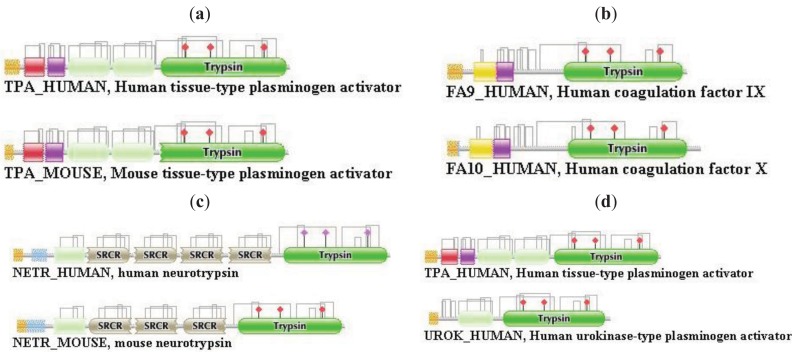
Types of homology of multidomain proteins: orthologs and paralogs. (**a**) Orthologous multidomain proteins with identical DA—human and mouse tissue plasminogen activator; (**b**) Paralogous multidomain proteins with identical DA—human factor 9 and human factor 10; (**c**) Orthologous multidomain proteins with different DA—human and mouse neurotrypsin; and (**d**) Paralogous multidomain proteins with different DA—human tPA and human urokinase.

**Figure 2:**
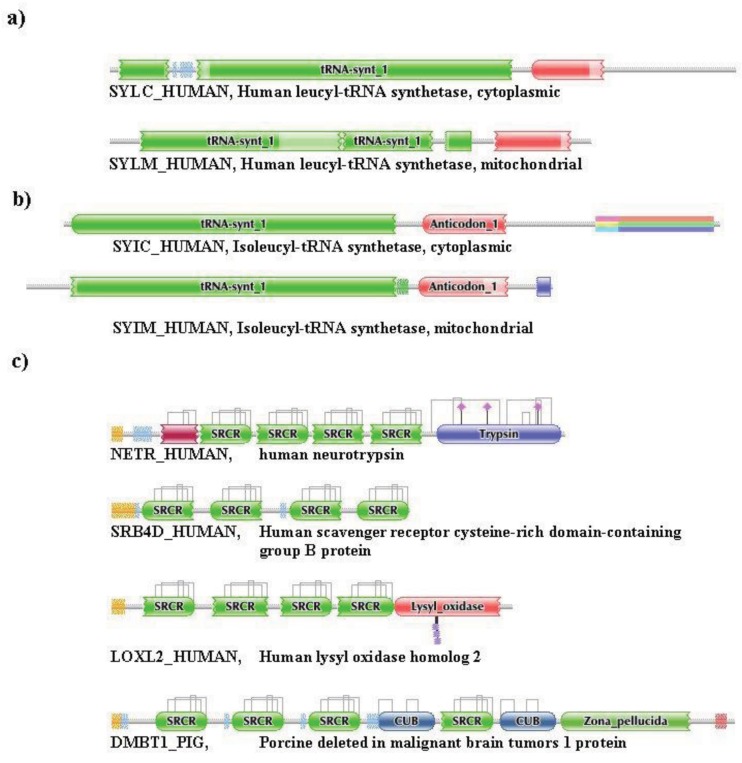
Types of homology of multidomain proteins: pseudoparalogs and epaktologs. (**a**) Pseudoparalogous multidomain proteins with identical DA—cytoplasmic and mitochondrial Leucyl-tRNA synthetase; (**b**) Pseudoparalogous multidomain proteins with different DA—cytoplasmic Leucyl-tRNA synthetase and mitochondrial Isoleucyl-tRNA synthetase; and (**c**) Epaktologous proteins sharing homologous domains—human neurotrypsin, human scavenger receptor cysteine-rich domain-containing group B protein, human lysyl oxidase homolog 2 and porcine deleted in malignant brain tumors 1 protein.

As illustrated in Figures 1 and 2, there exists an additional category of homologous multidomain proteins that are neither orthologous (they do not have the same common ancestor in the last common ancestor of the two species), nor paralogous (they do not derive from the same gene that was duplicated within a genome), nor pseudoparalogous (neither of them were acquired by inter-species HGT): their homology is the result of shuffling of mobile domains.

For example, human neurotrypsin, human scavenger receptor cysteine-rich domain-containing group B protein and human lysyl oxidase homolog 2 are related only in the sense that they all contain tandem SRCR domains (see Figure 2c).

Several attempts have been made to distinguish homology of multidomain proteins due to shuffling of mobile domains from other types of homology (orthology, paralogy, pseudoparalogy). Some authors have used a model in which two sequences were judged to be “homologous only if they are encoded by genes that share an ancestral locus” [7], thus excluding domain-shuffling based homology from the world of homologies. Fitch [5] recommended the use of the term ‘partial homology’ for cases where homology of two proteins does not hold for the entire length of both proteins, as suggested by Hillis [8]. The term ‘partial homology’, or its equivalent ‘local homology’ (as opposed to ‘global homology’) [9] do not grab the uniqueness of this type of homology since it is also valid for cases where two sequences are partially homologous but at the same time they are orthologous (or paralogous, pseudoparalogous), e.g., because one of them lost or gained a domain (see cases (c) and (d) in Figure 1 and case (b) in Figure 2.

Here we propose a new term for homologs that are neither orthologs nor paralogs/pseudoparalogs of each other yet they are related through the acquisition of homologous domains (see (c) in Figure 2. Since the basis of their homology is the import of homologous mobile domain(s), we suggest the term epaktology from the ancient Greek eπακτός, ‘imported’. Accordingly, in this manuscript we will refer to proteins that are related to each other only through acquisition of the same type of mobile domains as epaktologs.

Although many types of procedures exist for establishing orthology and paralogy of proteins, no simple solution exists for the distinction of closely related paralogs and closely related epaktologs. The significance of this problem may be illustrated by the fact that, in TreeFam [10,11], several trees for orthologous/paralagous multidomain proteins are contaminated with epaktologous multidomain proteins (for some examples see the sections ‘Results’ and ‘Discussion’ below and the accompanying paper [1].

**Figure 3:**
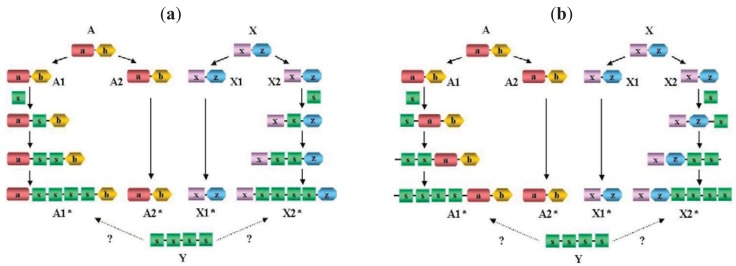
Domain Architecture evolution: consequences of confusing epaktology and paralogy. Proteins A and X are unrelated ancestral multidomain proteins. During evolution domain shuffling inserts the same domain-type (domain s) into orthologs of A and X proteins independently followed by tandem duplication of this domain, resulting in proteins A1* and X2* in an extant species (Species*). Overall sequence similarity score of proteins A1* and X2* may be so significant that A1* appears to be much more closely related to X2* than its true paralog (A2*), therefore, based on sequence similarity searches it may be concluded that X2* is the closest paralog of A1* (inferring. that A1* and X2* diverged from a common hypothetical ancestor Y) and that independent terminal gain of domains a,b and x,z occurred in the two lines leading to paralogs A1* and X2*. In contrast with this interpretation, A1* is a paralog of A2* (and X1* is a paralog of X2*) and independent gain and duplication of an s domain occurred in both lineages. Note the that although the two scenarios depicted in (a) and (b) differ in the actual events (internal *vs.* terminal insertion of domain s), if the epaktologs are treated as paralogs the conclusion will be similar in as much as the DA of A1* and X2* differ in terminal positions.

It must be emphasized that failure to separate paralogs from epaktologs may lead to serious errors in the interpretation of DA differences: if we compare the DA of two paralogs (separated by a single duplication event) we are likely to reconstruct the actual events (gain or loss of domains) that have occurred since the duplication of the ancestral gene, whereas if we compare two epaktologs we will be misled as to the evolutionary history of DA changes (Figure 3).

Proteins A and X, shown in Figure 3 represent unrelated ancestral multidomain proteins with domain architectures a-b and x-z, respectively. The a) panel illustrates the case where domain shuffling inserts the same domain-type (domain s) into orthologs of A and X proteins independently in an internal position, followed by tandem duplication of this domain, resulting in proteins A1* and X2* in an extant species with domain architectures a-s-s-s-s-b and x-s-s-s-s-z, respectively. (Note that A1* and X2* are epaktologs, their homology is due only to the independently imported s domain). Thanks to the tandem duplicated s domain sequence, the similarity of A1* and X2* may be so significant that A1* appears to be much more closely related to X2* than any of its paralogs (A2*), therefore, based on sequence similarity searches it might be concluded that X2* is the closest paralog of A1*. If we align and compare their domain architectures, we may be led to conclude that A1* and X2* diverged from a common hypothetical ancestor Y (with domain architecture of s-s-s-s) and that independent terminal gain of domains a,b and x,z occurred in the two lines leading to paralogs A1* and X2*. However, the truth is that A1 is a paralog of A1* (and X2* is a paralog of X1) and independent internal gain and duplication of an s domain occurred in both lineages.

The (b) panel of Figure 3 illustrates the case where domain shuffling inserts the same domain-type s, into orthologs of A and X proteins independently in terminal positions, followed by tandem duplication of this domain, resulting in proteins A1* and X2* in an extant species with domain architectures s-s-s-s-a-b and x-z-s-s-s-s, respectively. It should be pointed that although the two scenarios depicted in Figure 3a and Figure 3b differ in the actual events (internal *vs.* terminal insertion of domain s), if the epaktologs are treated as paralogs, the conclusion will be similar in as much as the DA of A1* and X2* differ in terminal positions.

Despite the problems that may be caused by confusing epaktologs and paralogs, the significance of this type of error in studies on DA evolution has not been explored. In the present work we used two types of paralogy-group construction procedures and monitored the impact of various parameters on the separation of true paralogs from epaktologs on Swiss-Prot entries of multidomain proteins with known evolutionary histories.

Our studies have shown that analysis of the structure of sequence similarity networks of multidomain proteins provides an efficient means for the separation of epaktologs and paralogs. We also demonstrated that failure to separate epaktologs and paralogs increases the apparent rate of DA change during protein evolution and falsifies the results by introducing a positional bias in favor of terminal over internal DA changes.

## 2. Results and Discussion

### 2.1. Datasets of Human Swiss-Prot Paralogs

We used two different approaches to define clusters of human paralogs: the first approach was based on intra-species comparison of human Swiss-Prot sequences, the second approach was based on comparison of human Swiss-Prot sequences with RefSeq proteomes of other Metazoa. The benefit of using alternative approaches is that they have different methodological and theoretical limitations.

As discussed below, intra-species comparison of human Swiss-Prot sequences benefits from the fact that the dataset is of high quality (essentially valid, complete, correct and nonredundant) and suffers only from the problem that it may be difficult to separate paralogs and epaktologs. Another limitation of this approach is that, in itself, it provides no information about the time of gene duplication(s) that gave rise to paralogs.

Conversely, comparison of human Swiss-Prot sequences with Refseq proteomes of other Metazoa suffers from the weaker quality of the target datasets (the RefSeq proteomes may be incomplete and/or redundant, some sequences may be non-valid, abnormal, incomplete or mispredicted), the problem that gene duplication and gene loss also occurred in the target genomes but benefits from the fact that comparison of clustering patterns obtained on different species provides information about the time of gene duplication(s) that gave rise to different human paralogs.

#### 2.1.1. Datasets of Paralogous Human Swiss-Prot Sequences Defined through Comparison of Human Swiss-Prot Entries

We have performed an all-against-all sequence comparison of human Swiss-Prot entries and the results of these sequence comparisons were ranked in the order of decreasing sequence similarity scores, including in this list only the top-scoring 1, 2, 3, …. 20 matches with e-values of <10^−5^, excluding self-matches. Distinct datasets containing the top-scoring one, two, … twenty sequences were created (hereafter referred to as TSS = 1, TSS = 2, …. TSS = 20 datasets).

In principle, in all-against-all sequence comparison of human Swiss-Prot entries, sequences that give significant matches with the query are either paralogs (results of gene duplication) or pseudoparalogs (reflecting interspecies horizontal gene transfer) or epaktologs (reflecting domain-shuffling) of the query.

Horizontal gene transfer (HGT) is known to have played a negligible role in the evolution of Metazoa therefore in the case of human sequences pseudoparalogy due to relatively recent HGT is insignificant [12]. Human pseudoparalogs were acquired by early eukaryotes via horizontal gene transfer from diverse bacteria; a substantial number of pseudoparalogous genes are derived from the mitochondrial endosymbiont [13]. The majority of these endosymbiont-derived pseudoparalogs are involved in translation, mostly aminoacyl-tRNA synthetases and ribosomal proteins, which are often represented by cytosolic and mitochondrial versions. Since the human cytoplasmic and mitochondrial pseudoparalogs are usually very distantly related (their common ancestor dates back to the time prior to the acquisition of mitochondria) the danger of confusing (relatively recent) paralogy with (ancient) pseudoparalogy is expected to be relatively low when a cut off of e-values of <10^−5^ is used.

This point may be illustrated by the case of SYLC_HUMAN (see Figure 2a). SYLC_HUMAN (Leucyl-tRNA synthetase, cytoplasmic) has very low sequence similarity (e-value > 10^−5^) with SYLM_HUMAN (Probable leucyl-tRNA synthetase, mitochondrial) although their domain architectures are similar: both proteins contain N-terminal tRNA-synt_1 and C-terminal Anticodon_1 domains.

In the next step we attempted to distinguish paralogs from epaktologs based on the rationale that the structure of sequence similarity networks is expected to be different for the two types of homologs [7]. We analyzed the structure of sequence similarity networks of paralogs with the Pajek software [14-16]. In these analyses we included increasing numbers of the top-scoring sequences (TSS = 1, TSS = 2, …. TSS = 20 datasets) and analyzed the structure of directed networks where nodes/vertices A, B, … Z correspond to individual sequences and an edge connects A and B if in the given dataset A (or B) is included among the top matching sequences of B (or A). We analyzed component structures of networks to define fully connected sub-networks (strong components) as well as weak components. In graph theory, a strong component of a network is defined as a subset of nodes such that for any pair of nodes u and v in the subset there is a path from u to v, whereas weak components contain all nodes which are connected, directly or indirectly, to each other by edges [17]. Based on the foregoing, it is expected that strong components of sequence similarity networks will primarily consist of paralogs, whereas in weak components, strong components of paralogs will be surrounded/connected by epaktologs.

##### 2.1.1.1. Human Swiss-Prot Protein Families Used to Monitor the Separation of Paralogs from Epaktologs

To monitor the reliability of this approach in separating paralogs from epaktologs we have selected members of several representative protein families where the evolutionary history is known. Some examples came from families known to be unaffected by domain-shuffling (such as TIMPs, Fructose-bisphosphate aldolases, see below), others came from families where domain-shuffling has played a major role in shaping DAs (‘paralogs and epaktologs’).

###### 2.1.1.1.1. Families with Paralogs and Orthologs Only

####### **TIMP2_HUMAN** (TreeFam tree TF317409)

All members of the TIMP protein-family have the same domain architecture (contain only Pfam A domain: TIMP) and apparently this family has never participated in domain-shuffling. The strong component defined for the dataset containing the first top-scoring sequence identified TIMP4_HUMAN as the closest paralog of TIMP2_HUMAN; the weak component contained all four known human paralogs TIMP1_HUMAN, TIMP2_HUMAN, TIMP3_HUMAN and TIMP4_HUMAN. As we increased the number of top-matching sequences to two top-scoring matches, both the strong and weak components contained all and only the four human TIMP paralogs and no further increase in the number of top-scoring sequences allowed had an influence on the contents of the strong or weak components. Note that the order in which paralogs were identified is consistent with the evolutionary history of TIMPs represented in TreeFam tree TF317409: the most recent gene duplication (in early vertebrates) gave rise to TIMP4/TIMP2 [18].

####### **ALDOA_HUMAN** (Treefam Tree TF314203)

All members of the Fructose-bisphosphate aldolase family have the same domain architecture (contain only Pfam A domain: Glycolytic). The strong component defined for the dataset containing the first top-scoring sequence identified ALDOC_HUMAN as the closest paralog of ALDOA_HUMAN; the weak component contained all three human paralogs ALDOA_HUMAN, ALDOB_HUMAN, ALDOC_HUMAN. As we increased the number of top-matching sequences to two top-scoring matches, both the strong and weak components contained all and only the three human aldolase paralogs and no further increase in the number of top-scoring sequences allowed had an influence on the contents of the strong or weak components. Note that the order in which paralogs were identified is consistent with the evolutionary history of Fructose-bisphosphate aldolases (see Treefam Tree TF314203).

In summary: in the case of proteins consisting of domains that apparently did not participate in domain-shuffling in the metazoan lineage (such as the TIMP and Glycolytic domains), paralog-identification with both strong and weak component analysis was efficient and reliable, irrespective of the number of top-scoring matches included in the analysis.

###### 2.1.1.1.2. Families with Paralogs, Orthologs and Epaktologs

Differences in the network structure properties of paralogs and epaktologs are illustrated by some selected multidomain proteins containing various types of mobile domains that were frequently involved in domain-shuffling events: TPA_HUMAN (contains mobile Pfam A domains: FN1, EGF and kringle; see Figure 1a), THRB_HUMAN (contains the mobile Pfam A domain: kringle), NETR_HUMAN (contains mobile Pfam A domains: kringle and SRCR; see Figure 2c), TSP2_HUMAN (contains mobile Pfam A modules: VWC, TSP_1, EGF), MYOC_HUMAN (contains mobile Pfam A module: OLF), MMP2_HUMAN (contains mobile Pfam A module: FN2), SE1L1_HUMAN (contains mobile Pfam A module: FN2) and AGRIN_HUMAN (contains mobile Pfam A modules: Kazal_1, Kazal_2, Laminin_EGF, SEA, EGF, Laminin_G_1). The motivation for this selection is that their evolutionary history has been characterized in some detail and that they differ in the number and degree of promiscuity of the mobile domains.

The evolutionary histories of the proteases of the blood coagulation and fibrinolytic cascades have been reconstructed from the evolutionary histories of their constituent domains [19-21], therefore we have selected several representatives from these families. TPA_HUMAN is a member of the plasminogen activator branch of fibrinolytic enzymes, THRB_HUMAN is a member of the coagulation factor branch, which, however, is unique in this branch in having acquired kringle-domains related to those of fibrinolytic enzymes by exon-shuffling [19]. NETR_HUMAN was also included in this analysis, since its kringle and protease-domains assign it to the large multigene family containing fibrinolytic proteases, but otherwise the domain organization of its non-catalytic region (several tandem SRCR domains) shows little similarity with these enzymes.

We have selected TSP2_HUMAN, a member of the thrombospondin family of proteins since the evolutionary histories of these proteins was analyzed in detail [22]. As to the number and promiscuity of constituent domains: MYOC_HUMAN, MMP2_HUMAN and SE1L1_HUMAN represent the case where there is a single mobile module-type with moderate versatility, whereas AGRIN_HUMAN represents the other extreme, with a large number of domains of high versatility [3].

####### **TPA_HUMAN** (TreeFam tree TF329901)

In the case of TPA_HUMAN the strong component for the dataset of the first top-scoring matches identified UROK_HUMAN as the closest paralog, the strong component for the dataset of the top two matches contained FA12_HUMAN, HGFA_HUMAN, HABP2_HUMAN, UROK_HUMAN and TPA_HUMAN, *i.e.*, known members of the plasminogen activator branch of proteases (TreeFam tree TF329901). This composition of the strong component did not change in the case of datasets containing the top three and top four matches, but when membership in the list of top-scoring sequences was increased to five, the strong component also included members of the plasminogen family (HGF_HUMAN, HGFL_HUMAN, MSTP9_HUMAN, APOA_HUMAN, PLMN_HUMAN) and coagulation factor families (FA7_HUMAN, FA9_HUMAN, FA10_HUMAN, THRB_HUMAN, PROZ_HUMAN, PROC_HUMAN), *i.e.*, more distant paralogs of TPA_HUMAN. TPA_HUMAN became incorporated into the Largest Connected Component (LCC) of the human sequence homology network (containing all types of multidomain proteins constructed from mobile domains) when >6 top scoring sequences were allowed to be included in the analyses.

**Figure 4:**
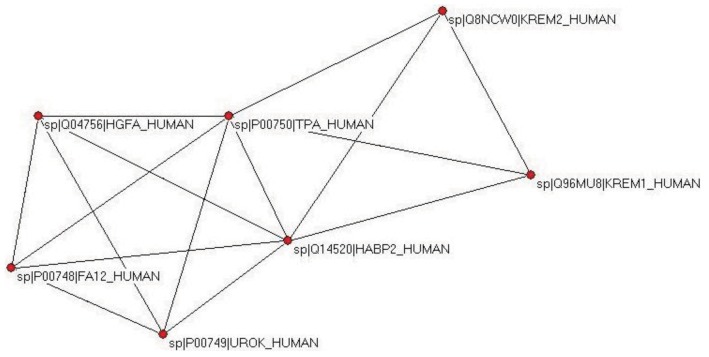
Cluster containing TPA_HUMAN defined by analysis of the sequence similarity network for TSS = 3. Note that strong component analysis identifies a cluster that contains only paralogs (TPA_HUMAN, UROK_HUMAN, FA12_HUMAN, HGFA_HUMAN, HABP2_HUMAN), whereas the cluster defined by weak component analysis also contains two epaktologs: KREM1_HUMAN and KREM2_HUMAN).

The weak component of the dataset of the first top-scoring matches also identified UROK_HUMAN as the closest paralog, but the datasets for the top two and three matches contained, in addition to valid paralogs (FA12_HUMAN, HGFA_HUMAN, HABP2_HUMAN, UROK_HUMAN, TPA_HUMAN), two epaktologs, KREM2_HUMAN and KREM1_HUMAN that are related to TPA_HUMAN only in that they also contain kringles, in addition to a WSC and a CUB domain (Figure 4); in TreeFam Kremen proteins are in family TF331319.

When the number of top matching sequences was increased to four, TPA_HUMAN was incorporated into the LCC, the giant component of the human sequence homology network containing the majority of multidomain proteins containing mobile domains. The DAs of TPA and its closest paralogs are illustrated in Figure S1.

####### **THRB_HUMAN** (TreeFam tree TF327329)

In the case of THRB_HUMAN, strong component analysis for the datasets of one, two and three top-scoring matches identified no paralogs, but the dataset of four top-scoring matches contained the known closest paralogs of the family of blood coagulation enzymes: FA7_HUMAN, THRB_HUMAN, FA10_HUMAN, PROC_HUMAN, FA9_HUMAN and PROZ_HUMAN (in harmony with TreeFam tree TF327329). When the number of top-scoring sequences was increased to five, the strong component also included members of the plasminogen-family (HGF_HUMAN, HGFL_HUMAN, MSTP9_HUMAN, APOA_HUMAN, PLMN_HUMAN) and plaminogen activator families (TPA_HUMAN, UROK_HUMAN, FA12_HUMAN, HABP2_HUMAN, HGFA_HUMAN), *i.e.*, more distant paralogs of THRB_HUMAN that also contain both kringle and Trypsin domains. The strong component also contained PROS_HUMAN, SHBG_HUMAN and GAS6_HUMAN. The similarity of PROS_HUMAN and GAS6_HUMAN with THRB_HUMAN is restricted to Gla- and EGF-domains; in TreeFam they are found in tree TF352157.

The weak component of the dataset of the first top-scoring matches assigned THRB_HUMAN to a cluster containing LPAL2_HUMAN, APOA_HUMAN, PLMN_HUMAN and PLGB_HUMAN, *i.e.*, distant, kringle-containing paralogs of THRB_HUMAN (but not the closest paralogs such as FA7_HUMAN, THRB_HUMAN, FA10_HUMAN, PROC_HUMAN, FA9_HUMAN and PROZ_HUMAN). Inclusion of two top-scoring matches increased the size of this cluster with the addition of PLGA_HUMAN, HGFL_HUMAN, HGF_HUMAN, MSTP9_HUMAN, whereas inclusion of three top-scoring matches resulted in the incorporation of THRB_HUMAN in the giant component containing the majority of multidomain proteins containing promiscuous domains. The DAs of THRB_HUMAN and its closest paralogs are illustrated in Figure S2.

####### **NETR_HUMAN** (TreeFam tree TF329295)

In the case of NETR_HUMAN, the strong component for the datasets of one and two top-scoring matches did not identify any candidates for paralogs, but the dataset of three, four, five top-scoring matches contained DMBT1_HUMAN, SRB4D_HUMAN, SRCRL_HUMAN, C163A_HUMAN and C163B_HUMAN. Note that these proteins are epaktologs of NETR_HUMAN: the high sequence similarity score comes from the presence of tandem SRCR domains in both sets of proteins (see also Figure 2c). Inclusion of six top-scoring matches resulted in the incorporation of NETR_HUMAN in the giant component of the network.

The weak component of the sequence similarity network of the first top-scoring matches identified numerous proteins containing tandem SRCR domains.

It must be emphasized that neither the strong nor the weak component analysis identified any of the true paralogs of this protease (members of the plasminogen-plasminogen activator family). This failure is due to the fact that sequence similarity is dominated by the four tandem SRCR domains of NETR_HUMAN, rather than its protease-domain (trypsin), therefore epaktologs (also containing tandem SRCR domains) were preferred over paralogs. Note that in Treefam (TreeFam tree TF329295) NETR-HUMAN, a member of the trypsin-family, is also assigned to the family of DMBT1-like proteins, illustrating the point that some TreeFam trees confuse epaktologs and paralogs (for further examples see the accompanying paper [1]). The DAs of NETR_HUMAN and some of its closest human epaktologs are also illustrated in Figure S3.

####### **TSP2_HUMAN** (TreeFam tree TF324917)

In the case of TSP2_HUMAN the strong component for the datasets of one and two top-scoring matches identified TSP1_HUMAN, its closest paralog. When the number of top-scoring sequences was increased to three, four, five, … eight, the strong component also included TSP3_HUMAN, COMP_HUMAN and TSP4_HUMAN, *i.e.*, all and only the known human paralogs of TSP2_HUMAN (that share the common TSP_C Pfam domain). Inclusion of more than eight top-scoring matches resulted in the incorporation of TSP2_HUMAN in the giant component (through the mobile TSP1 domain present in TSP1_HUMAN and TSP2_HUMAN).

The weak component of the dataset of the first top-scoring matches also identified TSP1_HUMAN, the closest paralog of TSP2_HUMAN. Inclusion of two top-scoring matches increased the size of this cluster to include TSP3_HUMAN, COMP_HUMAN, TSP4_HUMAN, *i.e.*, all and only the known human paralogs of TSP2_HUMAN, whereas inclusion of three top-scoring matches resulted in the incorporation of TSP2_HUMAN in the giant component. The DAs of TSP2_HUMAN and its paralogs are illustrated in Figure S4.

####### **MYOC_HUMAN** (TreeFam tree TF315964)

Human myocilin is a member of a human gene family that also contains gliomedin, olfactomedins and noelins; a common feature of these paralogs is that they all contain the Pfam A domain OLF.

In the case of strong component analysis of sequence similarity networks of TSS = 1, … TSS = 6 the protein was clustered only with paralogs OLM2B_HUMAN, NOE2_HUMAN, OLM2A_HUMAN, NOE3_HUMAN and NOE1_HUMAN. In the case of more than six top-scoring matches, the clusters also included epaktologs such as LPHN1_HUMAN, LPHN2_HUMAN and LPHN3_HUMAN that are related to MYOC_HUMAN only through the presence of an OLF domain. The latter proteins belong to the TreeFam tree TF351999 of G-protein coupled receptors. In the case of TSS = 15 MYOC_HUMAN was merged into the giant component of the sequence similarity network.

Weak component analysis of sequence similarity networks of TSS = 1 and TSS = 2 clustered MYOC_HUMAN with paralogs NOE2_HUMAN, NOE3_HUMAN, NOE1_HUMAN, OLFL1_HUMAN, OLFL3_HUMAN, OLFM4_HUMAN and GLDN_HUMAN. Inclusion of more than two top-scoring matches in the analysis resulted in its inclusion in the LCC of the network. The DAs of MYOC_HUMAN and its closests paralogs and epaktologs are illustrated in Figure S5.

####### **MMP2_HUMAN** (TreeFam tree TF315428)

Human matrix metalloprotease 2 is a member of a human gene family that consists of a large number of metalloproteases characterized by the presence of Peptidase_M10 Pfam domains. MMP2s (and MMP9s) are unique in this family in as much as they also contain three internal tandem FN2 domains acquired by exon-shuffling. Note that this represents a rare case when domains were inserted within domain boundaries: in this case the FN2 domain(s) were inserted within the boundaries of the Peptidase_M10 domain.

In the case of strong component analysis of sequence similarity networks of TSS = 1, … TSS = 8 the protein was clustered only with its closest paralog MMP9_HUMAN, inclusion of more than nine top-scoring matches resulted in its inclusion in the LCC of the network.

Weak component analysis of sequence similarity networks of TSS = 1 clustered it with MMP9_HUMAN and the epaktologs BSPH1_HUMAN, ESPB1_HUMAN that are related to MMP2_HUMAN only through the presence of tandem FN2 domains. BSPH1_HUMAN, ESPB1_HUMAN (present in TreeFam tree TF343543). In the case of TSS = 2 it was clustered with more distant paralogs (members of the MMP-family) as well as epaktologs BSPH1_HUMAN, ESPB1_HUMAN and FINC_HUMAN that are related to MMP2_HUMAN only through the presence of FN2 domains (FINC_HUMAN is present in TreeFam tree TF329915). The cluster also included the epaktologs VTNC_HUMAN and PRG4_HUMAN that are related to MMP2_HUMAN only through the presence of Hemopexin domains. VTNC_HUMAN and PRG4_HUMAN are present in TreeFam tree TF332780. Inclusion of more than two top-scoring matches in weak component analysis resulted in the inclusion of MMP2_HUMAN in the LCC of the network. The DAs of MMP2_HUMAN and its closests paralogs and epaktologs are illustrated in Figure S6.

####### **SE1L1_HUMAN** (TreeFam tree TF315257)

Human protein sel-1 homolog 1 is a member of a gene family characterized by the presence of several tandem repeats belonging to the Pfam A domain family Sel1. SE1L1 proteins are unique in this gene family in as much as they also contain a Pfam A domain, FN2.

In the case of strong component analysis of sequence similarity networks of TSS = 1, … TSS = 5, the protein was clustered only with its closest paralog SE1L2_HUMAN. Inclusion of six or seven top-scoring matches resulted in its clustering with epaktologs LY75_HUMAN, MRC1_HUMAN, PLA2R_HUMAN, MRC1L_HUMAN and MRC2_HUMAN that are related to SE1L1_HUMAN only in the presence of FN2 domains; these proteins are present in TreeFam tree TF316663. Strong component analysis of sequence similarity networks of more than seven top-scoring matches resulted in the inclusion of SE1L1_HUMAN in the large components of the network.

Weak component analysis of sequences similarity networks for TSS = 1 and TSS = 2 clustered SE1L1_HUMAN with its closest paralog, SE1L2_HUMAN. In the case of datasets of more than two top-scoring matches the protein is found in the LCC of the network. The DAs of SE1L1_HUMAN and its closests paralogs and epaktologs are illustrated in Figure S7.

####### **AGRIN_HUMAN** (TreeFam tree TF326548)

Human agrin is a member of a gene family that contains the multidomain proteins agrin, perlecan (PGBM_HUMAN) and pikachurin (EGFLA_HUMAN) characterized by the presence of multiple C-terminal Laminin_G domains. Agrins are unique in this family in as much as they also contain an Nta domain, a SEA domain and several tandem follistatin-related domains, identified by Pfam as domains Kazal_1 or Kazal_2 [23,24].

In the case of strong component analysis of sequence similarity network of TSS = 1 the protein was not clustered with any of its homologs. In the case of TSS = 2 and TSS = 3 it was clustered only with its closest paralog, EGFLA_HUMAN. Analyses of datasets for more than three top-scoring matches have shown that the protein is included in large components of the network that contain all three paralogs AGRIN_HUMAN, PGBM_HUMAN and EGFLA_HUMAN and numerous epaktologs of the latter proteins.

Weak component analysis of sequences similarity network for TSS = 1 clustered AGRIN_HUMAN with its paralogs PGBM_HUMAN and EGFLA_HUMAN and numerous epaktologs of these proteins. In the case of datasets of more than one top-scoring matches the protein is found in the LCC of the network. The DAs of AGRIN_HUMAN and its closests paralog are illustrated in Figure S8.

These observations confirm that:

(1) In the case of protein-families (domain-families) that did not participate in domain-shuffling, sequence similarity network analysis identifies clusters of paralogs with high sensitivity and specificity, irrespective of the number of top-scoring matches allowed or the mode of component analysis (see the examples of TIMP2_HUMAN and ALDOA_HUMAN).
(2) In the case of protein-families (domain-families) that did participate in domain shuffling, inclusion of a large number of top-scoring matches allowed (e.g., 20 top-scoring sequences) in the sequence similarity network results in the inclusion of the query sequence in the LCC of the network that contains the majority of multidomain proteins containing shuffled modules.
(3) There are significant differences in the sequence similarity network characterisics of paralogs and epaktologs. These differences may be exploited to separate paralogs from epaktologs, but separation depends on the choice of parameters (the number of top-scoring matches included in the analyses and the mode of component analysis). If we include only the closest paralogs (e.g., datasets TSS = 1, TSS = 2), and define strong components we may be confident that the sequences identified are paralogs (see examples of TPA_HUMAN, TSP2_HUMAN, MYOC_HUMAN, MMP2_HUMAN, SE1L1_HUMAN, AGRIN_HUMAN) but it may not be possible to identify paralogs (see example of THRB_HUMAN and NETR_HUMAN). If we include more members in the analyses (e.g., dataset TSS = 5), we may identify more distant paralogs, but there is an increasing danger that we include sequences that are not paralogs but epaktologs of the query sequence (see example of THRB_HUMAN and NETR_HUMAN).
(4) The example of NETR_HUMAN cautions that the structure of sequence similarity networks does not necessarily distinguish paralogs from epaktologs: if two unrelated proteins acquire the same type of domain independently (and that domain undergoes tandem duplications independently), the sequence similarity score of these epaktologs (due to the large segment of homologous repeats) may be greater than those with their paralogs (see Figure S3). The probability of confusing epaktologs and parlogs appears to be increased by tandem duplication of the mobile domains that mediate this confusion.

To illustrate the general validity of these conclusions we have analyzed the characteristics of the sequence similarity networks of human Swiss-Prot sequences defined through intraspecies comparisons.

##### 2.1.1.2. Characteristics of the Sequence Similarity Networks of Human Swiss-Prot Sequences Defined through Intraspecies Comparisons

We have defined strong and weak components of sequence similarity networks for datasets (TSS = 1, TSS = 2, TSS = 3, …. TSS = 20) and monitored the influence of the choice of parameters (the number of top-scoring matches included in the analyses and the mode of component analysis) on the number of components (number of ‘families’) and the size of components (size of ‘families’, number of related sequences in that cluster).

**Figure 5:**
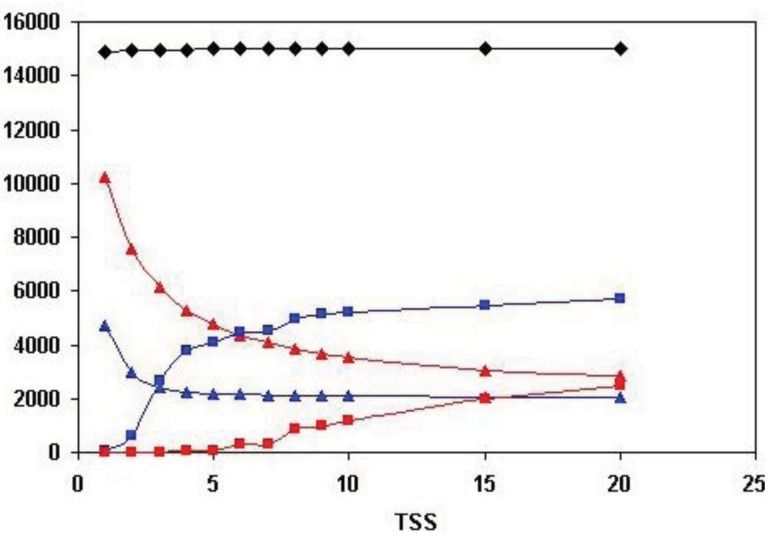
Analysis of sequence similarity networks of paralogous human proteins defined through all-against-all sequence comparison of human Swiss-Prot entries. The matches were ranked in the order of decreasing sequence similarity scores, including in this list only the top-scoring 1, 2, 3, …. 20 matches with e-values of <10^−5^, excluding self-matches. Datasets containing the top-scoring one, two… twenty sequences were created (TSS = 1, TSS = 2, …. TSS = 20 datasets) and the component structure of sequence similarity networks defined for these datasets was analyzed as described in the text. The numbers on the abscissa indicate the number of top-scoring matches included in the analyses (TSS = 1, …. TSS = 20). Black diamonds represent the number of human Swiss-Prot entries with at least one significant human Swiss-Prot homolog. Blue triangles represent the number of weak components, red triangles represent the number of strong components. Blue rectangles represent the number of sequences in the Largest Connected Component of weak component analysis, red rectangles represent the number of sequences in the Largest Connected Component of strong component analysis.

As shown in Figure 5, the total number of human Swiss-Prot sequences that are homologous (paralogous or epaktologous) with at least one human Swiss-Prot sequence (vertices in the network) is ∼15.000. Since the total number of human Swiss-Prot entries analyzed is 20.311, this indicates that ∼5000 human sequences are ‘solitary’, *i.e.*, they have no human homolog that give a significant similarity score at the cut-off value used (e-value < 10^−5^).

Figure 5 also illustrates that the number of both the weak and strong components decreases, whereas the size of the Largest Connected Component of weak component analysis increases sharply until the number of top-scoring sequences is increased to four (TSS = 4). The limiting value of the number of weak and strong components at high TSS values is ∼2000, *i.e.*, ∼2000 components are not incorporated into the LCCs. This observation indicates that ∼2000 human homology-clusters contain only domain-types that do not participate in domain-shuffling events (for example TIMPs and Fructose-bisphosphate aldolases, discussed above). The limiting value of the size of the LCC of weak component analysis is ∼6000, giving a rough estimate of the number of human proteins that belong to families that participated in domain-shuffling at least once.

A noteworthy difference between weak and strong component analysis is that the size of the Largest Connected Component of strong component analysis starts to increase only at higher numbers of top scoring sequences. Since LCC of weak component analysis sequesters the majority of protein-families that contain promiscuous domain-types, this difference confirms that strong components are much less affected by the problems of epaktology. The sharp increase in the number of entries in LCC of weak component analysis when the top three and four sequences are included in the analyses indicates that–in the case of weak components—this is the region where the boundary between paralogs and epaktologs is crossed for the majority of protein families containing promiscuous domains. Conversely, the fact that the size of LCC of strong component analysis is small (<100 proteins) for TSS = 1, TSS = 2, TSS = 3, TSS = 4 and TSS = 5 indicates that the homology-clusters of strong components are not significantly contaminated with epaktologs in the case of datasets containing just a few top-scoring sequences.

Analyses of strong components determined for the sequence similarity networks for TSS = 1, TSS = 2, TSS = 3, …. TSS = 20 revealed that in the case of TSS = 1, TSS = 2 the majority (61%, 59%, respectively) of the components with at least two sequences contained only sequences whose Swiss-Prot entry names were similar (at least the first three characters of the entry names were identical, e.g., SE1L1_HUMAN and SE1L2_HUMAN, F168A_HUMAN and F168B_HUMAN, GG12C_HUMAN and GG12H_HUMAN), consistent with the fact that the small clusters contain closely related paralogs. (It should be noted that non-similarity of entry names does not mean that they are non-paralogous, e.g., TPA_HUMAN and UROK_HUMAN; TSP4_HUMAN and COMP_HUMAN).

In view of these data we may conclude that, if we include only the closest paralogs (e.g., datasets TSS = 1, TSS = 2), and define strong components, we may be confident that the sequences identified are paralogs and not epaktologs.

#### 2.1.2. Datasets of Paralogous Human Proteins Defined through Interspecies Comparison of Human Swiss-Prot Entries with Refseq Sequences

The rationale of this approach is that if two human genes (genes A1 and A2) are derived from the ancestral gene A through gene duplication then they will give the highest sequence similarity score with the same extant A* sequence (the common ortholog of both human A1 and A2) in the proteomes of species that diverged from the human lineage before the duplication of gene A. This only occurs if gene A* was not lost in the latter species and if the complete, correct and non-redundant proteomes are known for all these species. If these conditions are valid then, and only then, comparison of human Swiss-Prot entries with complete proteomes of the various species that diverged prior to the duplication event will cluster paralogs A1 and A2.

Conversely, in the case of comparison of human Swiss-Prot, entries with complete proteomes of species that diverged from the human linaege after the duplication of gene A will give the greatest sequence similarity score with distinct extant sequences A1* and A2* (the orthologs of human A1 and A2, respectively) of the target proteomes, provided that gene A1* and/or gene A2* were not lost in the latter species and that the complete, correct and non-redundant proteomes are known for all these species. The change in clustering pattern may thus be used to define the time of gene duplication on the phylogenetic tree of the different taxonomic units.

It must be emphasized, however, that all the conditions formulated above may not be met by all the proteomes and this may cause problems in the interpretation of the data. First, gene duplication and gene loss is likely to have occurred in some of the lineages. Second, the proteomes may not be complete (mimicking gene loss) or may contain multiple predictions for the same gene (mimicking gene duplication) or may be mispredicted (depending on the type of error this may appear as gene loss and gene gain). Irrespective of whether absence of a sequence or presence of an additional copy of sequence is valid or an artifact, the conclusions might be misleading. In the case of ‘gene loss’ in the target genome, the query sequence may be misassigned to its most closely related paralog present in the species studied. Conversely, in the case of ‘gene duplication’ paralogous clusters may be split.

Distinct datasets containing paralogous clusters of human Swiss-Prot sequences were defined by blasting human Swiss-Prot entries against proteomes (Refseq datasets) of the following species: *Neurospora crassa, Saccharomyces cerevisiae, Schizosaccharomyces pombe, Monosiga brevicollis, Trichoplax adhaerens, Nematostella vectensis, Hydra magnipapillata, Caenorhabditis elegans, Caenorhabditis briggsae, Drosophila melanogaster, Drosophila pseudoobscura, Drosophila simulans, Anopheles gambiae, Apis mellifera, Strongylocentrotus purpuratus, Branchiostoma floridae, Ciona intestinalis, Danio rerio, Xenopus tropicalis, Gallus gallus, Mus musculus, Rattus norvegicus, Pongo pymaeus and Homo sapiens*. Note that RefSeq has much fewer *Xenopus tropicalis* entries than the number of genes predicted to be present in the frog genome. Conversely, RefSeq has a much higher number of entries for *Strongylocentrotus purpuratus, Mus musculus* and *Homo sapiens* than the number of genes predicted to be present in the corresponding genomes (see Table S1).

Clusters of homologous human Swiss-Prot entries were defined as sequences that gave the best match with the same entry in the given proteome; in these searches we used a cut-off value of e-value < 10^−5^.

Network properties of sequence similarity searches were analyzed with the Pajek software. In these analyses, for each entry of the target proteome (x_1_, x_2_, … x_N_), we listed human Swiss-Prot sequences (A, B, … Z) whose best match was the same (x_1_, x_2_, … x_N_) entry and analyzed the structure of directed networks where nodes/vertices A, B; …Z correspond to individual sequences and an edge connects A, B, … Z if in the given dataset their best match is the same entry in the target database. We have analyzed weak component structures of networks to define the number and size of components (clusters of homologs).

To monitor the performance of this approach we have selected members of several representative protein families where the evolutionary history is known. Here we illustrate our observations with the same proteins that we used above (section 2.1.1.1.2.) to get an insight into the separation of paralogs and epaktologs through the analysis of sequence similarity networks of human Swiss-Prot entries.

##### **TIMP2_HUMAN** (TreeFam tree TF317409)

The human proteome is known to have four human paralogs of the TIMP family, whereas the genomes of most invertebrate Metazoa are known to have a single TIMP protein. Comparison of human Swiss-Prot sequences with representative proteomes revealed that in the proteomes of *Trichoplax adhaerens, Drosophila melanogaster, Drosophila pseudoobscura, Drosophila simulans, Branchiostoma floridae* and *Ciona intestinalis* a single TIMP-related sequence clustered all four (and only the four) human TIMP paralogs, consistent with the view that the gene duplications giving rise to these paralogs occurred only in the vertebrate lineage. In the case of the mouse and human RefSeq proteomes orthologs (equivalents) of all four TIMP paralogs are present: these proteomes did not cluster the four TIMP paralogs.

In the case of *Nematostella vectensis, Hydra magnipapillata, Caenorhabditis elegans, Caenorhabditis briggsae* and *Strongylocentrotus purpuratus*, two or more TIMP-related sequences are present in the corresponding RefSeq datasets, splitting human TIMPs into separate clusters.

The influence of missing sequences may be illustrated by vertebrate species with less than complete proteomes (see Table S1). In the incomplete Refseq proteome of *Xenopus tropicalis* there are only two TIMPs that cluster human TIMPs into two different clusters. The Refseq proteome of *Gallus gallus* contains three TIMP-related sequences, one clusters TIMP1_HUMAN and TIMP2_HUMAN, the others are orthologs of TIMP3_HUMAN and TIMP4_HUMAN, respectively. Similarly, in the case of *Danio rerio* there is a clear ortholog of TIMP4_HUMAN, a second TIMP-related sequence clusters TIMP1_HUMAN, TIMP2_HUMAN and TIMP3_HUMAN.

##### **ALDOA_HUMAN** (Treefam Tree TF314203)

The human proteome is known to have three human paralogs of the fructose-bisphosphate aldolase family, ALDOA_HUMAN, ALDOB_HUMAN and ALDOC_HUMAN. Comparison of human Swiss-Prot sequences with representative proteomes revealed that in the proteomes of *Trichoplax adhaerens, Nematostella vectensis, Hydra magnipapillata, Caenorhabditis elegans, Caenorhabditis briggsae, Drosophila melanogaster, Drosophila simulans, Drosophila pseudoobscura, Strongylocentrotus purpuratus, Branchiostoma floridae* and *Ciona intestinalis* a single fructose-bisphosphate aldolase sequence clusters all three (and only the three) human fructose-bisphosphate aldolase paralogs. This is consistent with the fact that the gene duplications giving rise to these paralogs occurred early in the vertebrate lineage.

The genomes of most vertebrates studied (*Danio rerio, Xenopus tropicalis, Mus musculus*) contain orthologs of the three human paralog of fructose-bisphosphate aldolase, but a single aldolase-related sequence of the incomplete *Gallus gallus* proteome clusters the three human fructose-bisphosphate aldolase paralogs.

##### **TSP2_HUMAN** (TreeFam tree TF324917)

The human proteome is known to have five paralogs of the thrombospondin family [22]. Comparison of the human Swiss-Prot sequences with representative proteomes revealed that there is a single thrombospondin-related sequence in *Trichoplax adhaerens, Nematostella vectensis, Hydra magnipapillata, Drosophila melanogaster, Drosophila pseudoobscura, Drosophila simulans, Branchiostoma floridae* that cluster all five (and only the five) known human TSP paralogs (TSP1_HUMAN, TSP2_HUMAN, TSP3_HUMAN, TSP4_HUMAN and COMP_HUMAN).

In the case of *Strongylocentrotus purpuratus* and *Ciona intestinalis*, two different TSP-related sequences are present, splitting human TSPs into the two known subfamilies: TSP1_HUMAN/TSP2_HUMAN and TSP4_HUMAN/TSP3_HUMAN/COMP_HUMAN. This is consistent with the fact that the gene duplication, giving rise to these two main groups, occurred prior to the divergence of chordates from other deuterostomes.

*Danio rerio* and *Mus musculus* have orthologs for all five members of the thrombospondin family, therefore they do not cluster these paralogs. Since there are only two thrombospondins in the incomplete proteome of *Xenopus tropicalis*, this proteome defines two clusters of thrombospondin paralogs. In the case of *Gallus gallus* there are orthologs for COMP_HUMAN and TSP1_HUMAN, but TSP3_HUMAN and TSP4_HUMAN are clustered by a TSP-related sequence of chick. TSP2_HUMAN is clustered with an epaktolog, PROP_HUMAN (a protein that consists of tandem TSP_1 domains and is found in TreeFam tree TF315491), since an ortholog of the latter protein was missing from the chick RefSeq dataset.

##### **TPA_HUMAN** (TreeFam tree TF329901)

Comparison of human Swiss-Prot sequences with representative proteomes revealed that in the case of *Trichoplax adhaerens, Nematostella vectensis, Hydra magnipapillata, Caenorhabditis elegans, Caenorhabditis briggsae, Drosophila melanogaster, Drosophila pseudoobscura, Drosophila simulans* and *Strongylocentrotus purpuratus* TPA_HUMAN was present in large paralogous clusters containing trypsin-related proteases, reflecting a significant expansion of this gene family in vertebrates.

Comparison of human Swiss-Prot sequences with the RefSeq proteome of *Branchiostoma floridae* clustered TPA_HUMAN with its close paralogs (UROK_HUMAN, HGFA_HUMAN and HABP2_HUMAN), indicating that the plasminogen activator branch has separated from other proteases in early chordates. In the case of *Ciona intestinalis*, TPA_HUMAN was included in a larger cluster of proteases that, in addition to members of the plasminogen and plasminogen activator family (PLMN_HUMAN, FA12_HUMAN, UROK_HUMAN, TPA_HUMAN, APOA_HUMAN, HGF_HUMAN, HGFL_HUMAN, HABP2_HUMAN and MSTP9_HUMAN) also included THRB_HUMAN.

In the case of vertebrates *Danio rerio, Gallus gallus* and *Mus musculus*, TPA_HUMAN was not clustered with other proteases. This is consistent with the fact that the gene duplications giving rise to paralogous members of the plasminogen activator family occurred in early vertebrates. In contrast with other vertebrates, in the case of *Xenopus tropicalis* TPA_HUMAN was clustered with fibrinolytic proteases PLMN_HUMAN, FA12_HUMAN, UROK_HUMAN, HGF_HUMAN, HGFA_HUMAN and HABP2_HUMAN; this difference is due to the incompleteness of RefSeq dataset of this species.

##### **THRB_HUMAN** (TreeFam tree TF327329)

Comparison of human Swiss-Prot sequences with representative proteomes revealed that in the case of *Trichoplax adhaerens, Hydra magnipapillata, Caenorhabditis elegans, Caenorhabditis briggsae, Drosophila melanogaster, Drosophila pseudoobscura, Drosophila simulans* THRB_HUMAN was present in large paralogous clusters containing trypsin-type proteases, reflecting a significant expansion of this gene family in vertebrates.

*Strongylocentrotus purpuratus* clustered THRB_HUMAN with its paralog FA10_HUMAN, but *Ciona intestinalis* clustered it with members of the plasminogen and plasminogen activator family. In the case of vertebrates *Danio rerio, Gallus gallus* and *Mus musculus* THRB_HUMAN was not clustered with other proteases, which is consistent with the fact that the gene duplications, giving rise to paralogous proteases of the blood coagulation cascade, occurred in early vertebrates. In contrast with other vertebrates, in the case of *Xenopus tropicalis*, THRB_HUMAN was clustered with distant paralogs APOA_HUMAN and HGFL_HUMAN; this difference is due to the fragmentary nature of the RefSeq dataset for *Xenopus tropicalis*.

##### **NETR_HUMAN** (TreeFam tree TF329295)

Comparison of human Swiss-Prot sequences with proteomes of *Trichoplax adhaerens, Nematostella vectensis, Hydra magnipapillata, Drosophila melanogaster, Drosophila simulans Strongylocentrotus purpuratus* and *Branchiostoma floridae* revealed that in these cases NETR_HUMAN was clustered with its epaktologs: proteins containing tandem arrays of SRCR domains (e.g., SRCRL_HUMAN, DMBTL_HUMAN, CD6_HUMAN, LOXL3_HUMAN, LG3BP_HUMAN, SRCRM_HUMAN, LRAD2_HUMAN, C163A_HUMAN, SRB4D_HUMAN, LOXL4_HUMAN, C163B_HUMAN, DMBT1_HUMAN).

Interestingly, in the case of *Caenorhabditis elegans, Caenorhabditis briggsae*, NETR_HUMAN was clustered with paralogous trypsin-type proteases. The reason why paralogs were preferred over epaktologs in this case is that these nematode genomes have very few proteins with tandem SRCR domains (see Pfam database). Similarly, our observation that *Ciona intestinalis* clustered NETR_HUMAN with proteases (rather than with SRCR-containing proteins) is due to the fact that there are few proteins in Urochordates that contain tandem SRCRC domains (see Pfam database).

In the case of the vertebrates *Danio rerio, Gallus gallus* and *Mus musculus*, NETR_HUMAN was not clustered with other proteins. This indicates the presence of orthologs in these genomes, which is consistent with the view that the gene duplication giving rise to neurotrypsin gene occurred in early vertebrates. In contrast with other vertebrates, in the case of *Xenopus tropicalis*, NETR_HUMAN was clustered with epaktologous proteins with tandem arrays of SRCR domains (DMBT1_HUMAN etc.); this difference is due to the fragmentary nature of the RefSeq dataset for this species.

##### **MYOC_HUMAN** (TreeFam tree TF315964)

Human myocilin is a member of a human gene family that also contains gliomedin, olfactomedins and noelins. A common feature of these paralogs is that they all contain the Pfam A domain OLF. Comparison of human Swiss-Prot sequences with proteomes of *Trichoplax adhaerens, Caenorhabditis, Drosophila, Strongylocentrotus purpratus, Branchiostoma floridae* and *Ciona intestinalis* revealed that the protein was clustered with known paralogs, whereas, in the case of *Danio rerio, Gallus gallus, Mus musculus* RefSeq proteomes there are unique matches (1:1 orthologs) of MYOC_HUMAN. These observations concur with the fact that the gene duplication that gave rise to myocilin occurred in early vertebrates.

In contrast with other vertebrates, in the case of the incomplete proteome of *Xenopus tropicalis*, MYOC_HUMAN was clustered with a paralog, NOE2_HUMAN and an epaktolog, LPHN3_HUMAN. The latter protein, belonging to the TreeFam tree TF351999 of G-protein coupled receptors, is related to MYOC_HUMAN only through the presence of an OLF domain.

##### **MMP2_HUMAN** (TreeFam tree TF315428)

Human matrix metalloprotease 2 is a member of a human gene family that consists of a large number of metalloproteases characterized by the presence of Peptidase_M10 domains. MMP2s (and MMP9s) are unique in this family in as much as they also contain three tandem FN2 domains acquired by exon-shuffling. Comparison of human Swiss-Prot sequences with proteomes of *Trichoplax adharens, Nematostella vectensis, Hydra magnipapillata, Caenorhabditis, Drosophila, Strongylocentrotus purpuratus, Branchiostoma floridae* and *Ciona intestinalis* revealed that, in these cases, the protein was clustered with other metalloproteases, whereas in the case of *Danio rerio, Gallus gallus, Mus musculus* RefSeq proteomes there are unique matches (1:1 orthologs) of MMP2_HUMAN. These observations are in harmony with the fact that the gene duplication that gave rise to matrix metalloprotease 2 occurred in early vertebrates.

In contrast with other vertebrates, in the case of the incomplete proteome of *Xenopus tropicalis* MMP2_HUMAN was clustered with an epaktolog, SE1L1_HUMAN. The latter protein, belonging to TreeFam tree TF315257 of sel-1 homolog precursor proteins is related to MMP2_HUMAN only through the presence of an FN2 domain.

##### **SE1L1_HUMAN** (TreeFam tree TF315257)

Human protein sel-1 homolog 1 is a member of a gene family characterized by the presence of several in tandem repeats belonging to the Pfam A domain family Sel1. SE1L1 proteins are unique in this gene family in as much as they also contain a Pfam A domain, FN2. Comparison of human Swiss-Prot sequences with proteomes of *Trichoplax adharens, Nematostella vectensis, Hydra magnipapillata, Caenorhabditis, Drosophila, Strongylocentrotus purpuratus, Branchiostoma floridae* and *Ciona intestinalis* revealed that in these cases the protein was clustered with other sel-1 paralogs (SE1L2_HUMAN, LR2BP_HUMAN). In the case of *Danio rerio, Gallus gallus* it was clustered with SE1L2_HUMAN, whereas in the case of *Mus musculus* there was a unique match (1:1 ortholog) of SE1L1_HUMAN. These observations are in harmony with the fact that the gene duplication that gave rise to LR2BP_HUMAN and SE1L1_HUMAN/SE1L2_HUMAN occurred in early vertebrates.

In contrast with other vertebrates, in the case of the incomplete proteome of *Xenopus tropicalis*, SE1L1_HUMAN was clustered with an epaktolog, MMP2_HUMAN. The latter protein is related to SE1L1_HUMAN only through the presence of an FN2 domain.

##### **AGRIN_HUMAN** (TreeFam tree TF326548)

Human agrin is a member of a gene family that contains the multidomain proteins agrin, perlecan (PGBM_HUMAN) and pikachurin (EGFLA_HUMAN) characterized by the presence of multiple C-terminal Laminin_G domains. Agrins are unique in this family in as much as they also contain several tandem follistatin-related domains, identified by Pfam as domains Kazal_1 or Kazal_2 [23,24]. Comparison of human Swiss-Prot sequences with proteomes of various species revealed that AGRIN_HUMAN was prone to be clustered with epaktologs that also contain Kazal domains or Laminin_G domains. As a typical example we may mention the case of *Trichoplax where*, in addition to the paralog EGFLA_HUMAN, agrin was clustered with epaktologs that contain Kazal domains (ISK7_HUMAN, FSTL1_HUMAN) and epaktologs that also contain Laminin_G domains (CSPG4_HUMAN, NRX2A_HUMAN, NRX2B_HUMAN, NRX1A_HUMAN, NRX3A_HUMAN). Note that, in TreeFam, these epaktologs are represented in distinct trees; tree TF321302 (neurexins), tree TF316876 (CSPG4), tree TF106409 (follistatins) and tree TF106457 (Kazal type peptidase inhibitors).

In the case of *Nematostella*, AGRIN_HUMAN was clustered with epaktologous neurexins (NRX2B_HUMAN, EGFLA_HUMAN, NRX2A_HUMAN, NRX3A_HUMAN), whereas in the case of *Hydra* it was clustered with steroid-binding globulin, SHBG_HUMAN. SHBG_HUMAN, containing two Laminin_G domains, is represented in TreeFam tree TF334367. In the case of *Caenorhabditis*, AGRIN_HUMAN was clustered with epaktologous follistatin-related proteins such as FSTL3_HUMAN, FST_HUMAN, TEFF1_HUMAN, TEFF2_HUMAN and Kazal type peptidase inhibitors such as ISK5_HUMAN, ISK1_HUMAN, ISK8_HUMAN, ISK2_HUMAN, ISK53_HUMAN, ISK6_HUMAN. Note that TEFF1_HUMAN and TEFF2_HUMAN are represented in TreeFam tree TF330868. In the case of *Strongylocentrotus*, AGRIN_HUMAN was clustered with epaktologous follistatin-related proteins and Kazal type peptidase inhibitors (ISK4_HUMAN, ISK1_HUMAN, ISK53_HUMAN, TEFF1_HUMAN, ISK5_HUMAN, TEFF2_HUMAN). Similarly, Branchiostoma clustered AGRIN_HUMAN with ISK1_HUMAN, ISK52_HUMAN, TEFF1_HUMAN and TEFF2_HUMAN. In the case of *Ciona*, AGRIN_HUMAN was a member of a larger cluster of paralogous (EGFLA_HUMAN) and epaktologous proteins containing Kazal domains (ISK4_HUMAN, FSTL3_HUMAN, ISK1_HUMAN, FST_HUMAN, ISK52_HUMAN, ISK6_HUMAN, TEFF1_HUMAN, TEFF2_HUMAN). The DAs of AGRIN_HUMAN and some of its epasktologs are compared in Figure S9.

In the case of Danio rerio AGRIN_HUMAN was clustered with Kazal type protease inhibitors (ISK1_HUMAN, ISK7_HUMAN, ISK5_HUMAN). In the *Gallus gallus* and *Mus musculus* RefSeq proteomes there are unique matches (1:1 orthologs) of AGRIN_HUMAN. In the case of *Xenopus tropicalis*, AGRIN_HUMAN was clustered with Laminin_G containing epaktologs SLIT1_HUMAN, SLIT2_HUMAN (represented in TreeFam tree TF332887) due to the fact that RefSeq proteome of *X. tropicalis* is incomplete.

These observations suggest that:

(1) The majority of human paralogs that arose in the vertebrate lineage are correctly clustered in sequence similarity networks defined by complete invertebrate proteomes (see the examples of TIMP2_HUMAN, ALDOA_HUMAN, TPA_HUMAN, TSP2_HUMAN, THRB_HUMAN, MYOC_HUMAN, SE1L1_HUMAN, MMP2_HUMAN).
(2) Clusters are usually not contaminated with epaktologs, provided that the reference sequence is complete. However, if the RefSeq proteome used to define clusters of human paralogs is incomplete (e.g., the proteome of *Xenopus tropocalis*), the paralogous clusters are likely to be contaminated with epaktologs (see the cases of NETR_HUMAN, MYOC_HUMAN, MMP2_HUMAN, AGRIN_HUMAN).
(3) The examples of NETR_HUMAN and AGRIN_HUMAN caution that the inter-species comparison approach is also likely to confuse epaktology if unrelated proteins acquire the same type of domain independently and that domain undergoes tandem duplications independently. In such cases, the sequence similarity score of these epaktologs (due to the large segments of homologous repeats) may be greater than those with their paralogs (see Figure 3). In general, the probability of confusion of epaktologs with paralogs is increased by the presence of tandem arrays of repeated Pfam A domains. Note that in the examples discussed above, the mobile domains mediating epaktology (TSP1-, FN2-, SRCR-, Kazal_1-, Kazal_2-, Laminin_G1- and LamininG2-domains) are duplicated.

To assess the influence of these factors on a more quantitative basis we have analyzed the characteristics of the sequence similarity networks of human Swiss-Prot sequences defined through comparison with RefSeq sequences of representative Metazoan species using the Pajek software.

##### 2.1.2.1. Characteristics of the Sequence Similarity Networks of Human Swiss-Prot Sequences Defined through Interspecies Comparison with Refseq Sequences

We have defined the component structures of sequence similarity networks for datasets obtained by comparison of human Swiss-Prot sequences with proteomes of various species and monitored the influence of the evolutionary distance of the query species and target species on the number of components (number of ‘families’ with at least two members) and the size of components (size of ‘families’).

**Figure 6:**
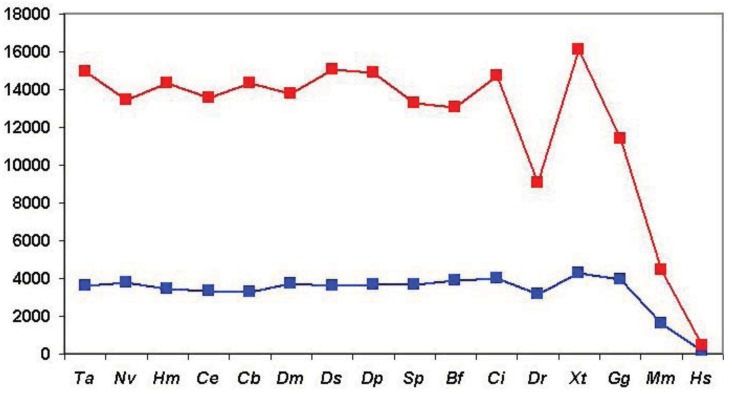
Analysis of sequence similarity networks of paralogous human proteins defined through comparison of human Swiss-Prot entries with proteomes of Metazoanspecies. Clusters of homologous human Swiss-Prot entries were defined as sequences that gave the best match with the same entry in the given proteome using a cut-off value of e < 10^−5^. The species are listed in the order of decreasing evolutionary distance from *Homo sapiens*, thus the abscissa has a time-dimension but their distance is not drawn to scale. Blue rectangles represent the number of components (homologous clusters), red rectangles represent the number of human Swiss-Prot entries that are clustered by sequences of the target genome, *i.e.*, that have at least one human paralog. Abbreviations on the abscissa: *Ta - Trichoplax adhaerens, Nv - Nematostella vectensis, Hm - Hydra magnipapillata, Ce -Caenorhabditis elegans, Cb -Caenorhabditis briggsae, Dm -Drosophila melanogaster, Dp -Drosophila pseudoobscura, Ds - Drosophila simulans, Sp - Strongylocentrotus purpuratus, Bf - Branchiostoma floridae, Ci - Ciona intestinalis, Dr - Danio rerio, Xt - Xenopus tropicalis, Gg - Gallus gallus, Mm - Mus musculus, Hs -Homo sapiens*.

As shown in Figure 6 (and Table S1), when we clustered human homologs with various invertebrate genomes (including invertebrate chordates) ∼14,500 human Swiss-Prot sequences had at least one homolog and these homologs were clustered into ∼3600 components. (Note that comparison of human/human Swiss-Prot entries yielded ∼15.000 as the number of human sequences that have at least one human homolog; see section 2.1.1.2). The somewhat lower number of human Swiss-Prot sequences clustered by invertebrate genomes reflects the fact that virtual or real ‘gene duplications’ affect the proteomes of invertebrate species. It is noteworthy in this respect that the lower values are observed in the case of redundant RefSeq proteomes where coverage is >1.0, whereas the higher values are observed in the case of RefSeq proteomes where coverage is <1.0 (see Table S1). Nevertheless, the observation that the vast majority (97%) of human Swiss-Prot paralogs clustered by comparison of human Swiss-Prot sequences are also clustered by invertebrate proteomes (including invertebrate chordates) confirms that the majority of human paralogs arose in the vertebrate lineage.

Consistent with this view, when human Swiss-Prot sequences were clustered with RefSeq proteomes of *Danio rerio* and *Mus musculus*, we observed a decrease in the number of components and the number of human sequences clustered in these components (see Table S1 and Figure 6), indicating that many of the paralogs arose as a result of gene duplication in early vertebrates. The fact that this decrease is not observed in the case of the proteomes of *Xenopus tropicalis* and *Gallus gallus* reflects the fact that these proteomes are incomplete.

The observation that ∼9000 human Swiss-Prot sequences are clustered in the sequence similarity network defined by the *Danio rerio* RefSeq proteome (Table S1) indicates that gene duplications that occurred in the human lineage since its divergence from the teleost lineage account for ∼9000 of the ∼15,000 human paralogs. This increase in the number of paralogs is primarily due to expansion of a limited number of gene families. Analyses of the clusters of human paralogs defined by the Danio rerio Refseq dataset revealed that the largest cluster (cluster size 304) contained olfactory receptors, consistent with a major expansion of this family in mammals [25].

Similarly, the observation that, in the case of *Mus musculus*, 1628 components cluster 4419 human sequences (Table S1) indicates that gene duplications occurred in a limited number of gene-families in the human lineage since its divergence from the rodent lineage. Inspection of the clusters of paralogs defined by the mouse RefSeq dataset revealed that the largest cluster (cluster size 45) contained zinc finger proteins of the zf-C2H2-family consistent with a major expansion of this family in primates [26].

The influence of incomplete proteomes on paralog identification is best illustrated by the case of *Xenopus tropicalis* and *Gallus gallus* proteomes: as a result of missing sequences some human sequences do not find their orthologs as their best matches and will instead be clustered with their paralogs or epaktologs, thus overestimating the number of human paralogs with a single common ancestor in the target proteome. For example, since many orthologs are missing from the incomplete RefSeq datasets of *Xenopus tropicalis* and *Gallus gallus* the total number of apparent human Swiss-Prot ‘paralogs’ is increased to 16,136 and 11,411, respectively, as opposed to the 9034 defined by the RefSeq dataset of *Danio rerio* (see Table S1 and Figure 6).

The influence of dataset errors of the RefSeq database is most obvious in the case of comparison of human Swiss-Prot entries with human RefSeq dataset. If both datasets were complete, correct and non-redundant there should be 1:1 correspondence between Swiss-Prot and RefSeq entries. As shown in Table S1 and Figure 6, 419 Swiss-Prot entries were clustered by 193 RefSeq sequences. A survey of the clusters of human Swiss-Prot paralogs defined by the *Homo sapiens* Refseq dataset revealed that the majority of clusters contained sequences whose entry names were similar (at least the first three characters were the same, indicating that one of the paralogous sequences was not represented in RefSeq). Another major characteristic of these clusters is that one or more members belong to the category of ‘putative uncharacterized proteins’ whose existence is uncertain (products of dubious predictions). Such Swiss-Proteins are frequently distinguished by entry names of the type Yxxx_HUMAN.

In summary: our analyses have shown that paralogous human Swiss-Prot proteins that arose in the vertebrate lineage may be reliably clustered with complete, nonredundant invertebrate proteomes. As illustrated by the case of *Xenopus tropicalis* proteome, the problem of contamination of paralogous clusters with epaktologs is significant in the case of incomplete proteomes.

### 2.2. Comparison of the DA of Paralogous Human Swiss-Prot Proteins

#### 2.2.1. Comparison of the DA of Paralogous Human Swiss-Prot Proteins Defined through Intraspecies Comparisons

To monitor the influence of the contamination of paralog-clusters with epaktologs we have determined DA differences in paralogous clusters defined for different number of top scoring sequences based on strong component analysis of sequence similarity networks of human Swiss-Prot sequences. Our expectation was that contamination of paralogous clusters with epaktologs should be reflected in an increased rate of DA difference. This expectation is based on the fact that close paralogs are likely to have identical or very similar DA (differing in a low number of domains), whereas epaktologs necessarily differ in DA and their difference may involve multiple domains (see Figure 2c, Figure 3 and the examples discussed above).

Our analyses have shown that at TSS = 1 (when only the most closely related paralogs are clustered), 92% of the clusters are homogeneous in as much as they contain only sequences with identical DA but this value decreases to 87%, 83%, 81%, 80%, 79% and 79% for TSS = 2, TSS = 3, TSS = 4, TSS = 5, TSS = 6 and TSS = 7, respectively (*i.e.*, when more distant paralogs are also clustered). In other words, in the case of TSS = 4, … TSS = 7, ∼ 20% of the clusters contain sequences with different DAs; these are the clusters that may also contain epaktologs.

**Figure 7:**
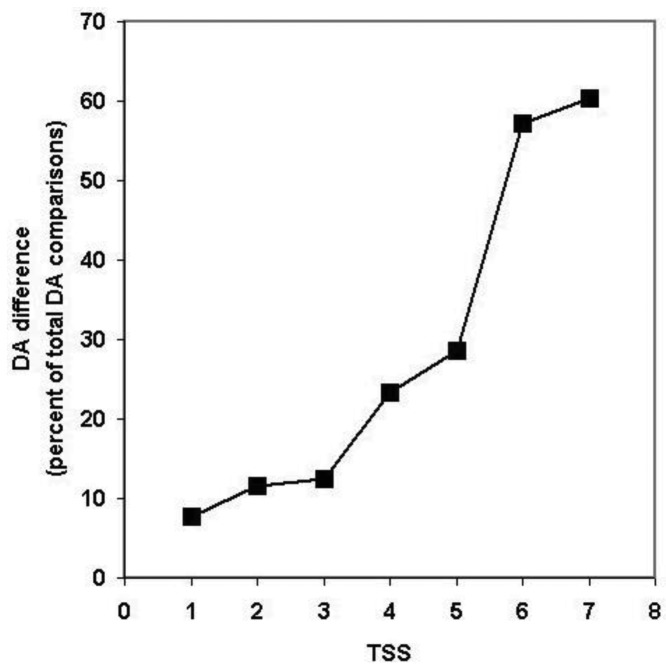
Analysis of the DA of clusters defined by strong component analysis of sequence similarity networks of human Swiss-Prot sequences. The numbers on the abscissa indicate the number of top-scoring matches included in the analyses (TSS = 1, …. TSS = 7) used to define paralogous clusters. The values of the ordinate show the percent of DA comparisons within clusters where the pairs compared differ in DA. (Since the number of pair-wise comparisons and computational time increased exponentially with the increase of TSS values, the figure shows only data for TSS = 1−TSS = 7).

Since, in the above classification, the size of the clusters is hidden, no distinction is made between a cluster with just a pair of sequences with identical DA and a cluster with 50 sequences of identical DAs (they are both clusters with identical DAs). Similarly, a cluster with five sequences each of which differs in DA and a cluster with 50 members just one of which differs from the others in DA, will equally qualify as a cluster with different DA. To estimate the degree of DA heterogeneity within clusters, we have calculated the percent of all-against-all DA comparisons that show a difference in DA (Table S2 and Figure 7).

Comparison of the DA of pairs defined for TSS = 1, TSS = 2, … TSS = 7 revealed (Figure 7, Table S2) that the percent of comparisons that gave different DA is ∼ 8% when just the closest paralogs (TSS = 1) are compared and the proportion of DA differences increases to 12%, 13%, 23% and 29% with the inclusion of more distant paralogs (TSS = 2, 3, 4, 5, respectively). The proportion of comparisons that gave DA difference increases sharply when clusters defined by more than five top-scoring sequences are analyzed (e.g., TSS = 6, TSS = 7), reflecting the fact that this is the point where the paralogous clusters become contaminated with epaktologs (see also Figure 5).

It should be noted that, even for the closest paralogs (TSS = 1), the rate of DA difference (8%) is higher than those observed when DAs of human Swiss-Prot proteins were compared with those of their vertebrate Swiss-Prot orthologs (<3% DA differences; see accompanying paper by [4]). Since the majority of paralogs present in the different clusters resulted from gene duplications in the vertebrate lineage (see section 2.1.2.1), this difference suggests that the rate of DA change is higher in paralogs than in orthologs.

**Figure 8:**
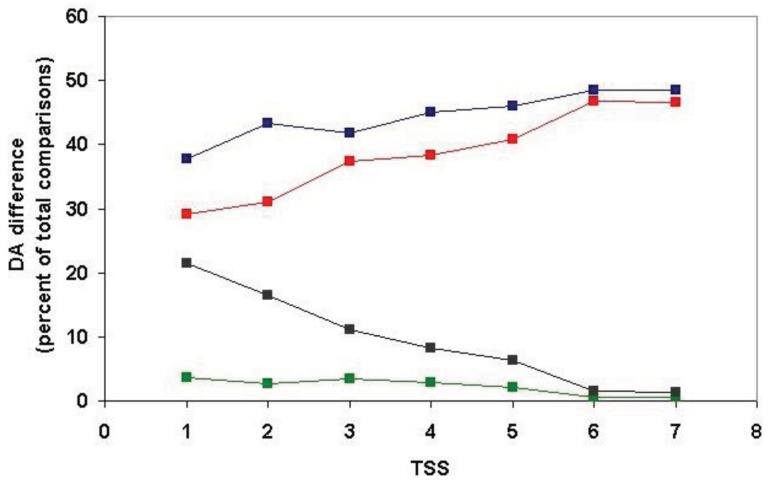
Analysis of the positional distribution of DA differences in clusters defined by strong component analysis of sequence similarity networks of human Swiss-Prot sequences. The numbers on the abscissa indicate the number of top-scoring matches included in the analyses (TSS = 1, …. TSS = 7) used to define paralogous clusters. N-terminal differences (blue rectangles), C-terminal differences (red rectangles), internal differences (green rectangles), tandem duplications (black rectangles).

Analysis of the positional distribution of DA differences within clusters defined for TSS = 1, TSS = 2, TSS = 3, … TSS = 7 revealed that the proportion of unassigned DA is negligible (<5%) for all datasets and that terminal domain differences always exceed internal differences (Table S3 and Figure 8).

For TSS = 1 clusters (containing just the closest paralogs and least likely to be contaminated by epaktologs) N-terminal DA changes account for the greatest proportion of DA differences (37.7%), C-terminal differences account for 29.1% of differences, 21.4% of the differences are of the duplication type and only 3.7% of DA changes belong to the internal category. Inclusion of more and more distant paralogs (and epaktologs) in the clusters increase the proportion of terminal DA differences; at TSS = 6 and TSS = 7 these approach the limiting value of 50–50%. Conversely, inclusion of more distant paralogs (and epaktologs) in the clusters results in a decrease in the proportion of internal DA differences and internal duplication at TSS = 6 and TSS = 7 their value drops to practically 0%. The explanation for these changes in pattern at TSS = 6 is that this is the point where contamination of paralogous clusters with epaktologs becomes significant (see section 2.1.1.2). As pointed out in the Introduction, contamination of clusters with epaktologs introduces a strong bias in favor terminal changes over internal changes and DA differences of the duplication type are likely to be suppressed since epaktologous pairs are typically aligned through shared tandem duplicated domains (see Figure 2c and Figure 3).

Irrespective of the problems caused by epaktologs, the fact remains that, even for TSS = 1 clusters (containing just the closest paralogs), terminal DA differences account for the majority of difference, whereas only 3.7% of DA changes belong to the internal category (Table S3). To decide whether this reflects a true difference in the probability of terminal changes or reflects the high proportion of one-domain ↔ two-domain transitions (type 1 transitions) in the paralogous datasets, we had to analyze the positional distribution separately for type 1, type 2 and type 3 transitions. As discussed in the accompanying paper [4], in the case of type 1 transitions, DA change by definition can only be classified as terminal (e.g., A ↔ AB or A ↔ BA).

**Figure 9:**
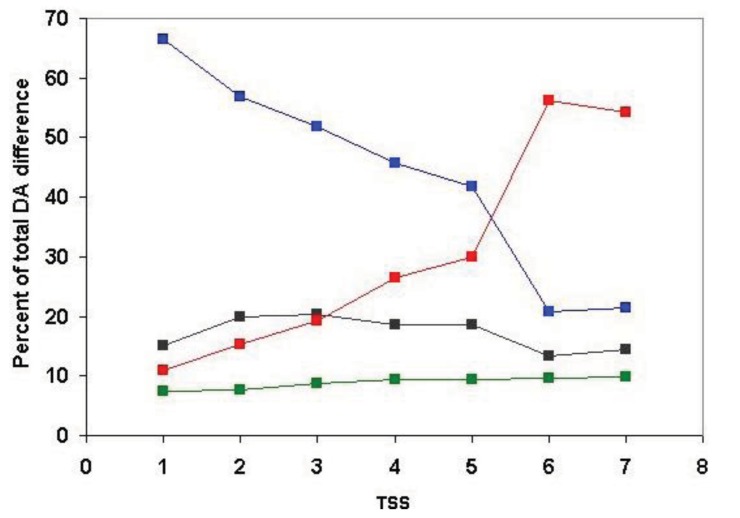
Analysis of the relative frequency of homologous pairs of human Swiss-Prot sequences that differ in the number of domains by 1, 2, 3, … N domains in clusters defined by strong component analysis of sequence similarity networks of human Swiss-Prot sequences. Note that, in the case of close paralogs (TSS = 1, TSS = 2), the majority of pairs differ in a single domain (blue rectangle) and a small proportion of homologs differs in 2 (black rectangle), 3 (green rectangle) or ≥4 domains (red rectangle). Inclusion of more distant paralogs had little influence on the proportion of pairs that differ in 2 or 3 domains, however, a sharp increase in the proportion of DA changes involving ≥4 domains is observed when more than 5 top-scoring matches are included in the analysis. The numbers on the abscissa indicate the number of top-scoring matches included in the analyses used to define paralogous clusters.

Analysis of the relative frequency of homologous pairs of human Swiss-Prot sequences that differ in the number of domains by 1, 2, 3, … N domains revealed that—for all datasets—pairs differed most frequently in single domains. Pairs that differed in 2 domains, 3 domains … N domains were increasingly less frequent (Table S4). However, the datasets of TSS = 1, TSS = 2, …. TSS = 7 showed significant differences in the relative frequency of homologous pairs of human Swiss-Prot sequences that differ in the number of domains by 1, 2, 3, … N domains: for example, with the increase of the number of top matches the proportion of DA differences involving at least four domains increased (see Table S4 and Figure 9.). The sharp increase in the proportion of this category observed when more than 5 top-scoring matches are included in the analysis is consistent with the notion that this is the point when clusters are significantly contaminated with epaktologs. (Note that all the contaminating epaktologs discussed in section 2.1 differ in DA from those of true paralogs in several domains).

To suppress the impact of epaktologs, we have analyzed only DA changes involving single-domain changes. Within this category, inclusion of more and more distant paralogs in the clusters had little influence on the relative proportion of type 1 transitions (43–50%), type 2 transitions (21–26%) and type 3 transitions (25–35%; see Table S5).

**Figure 10:**
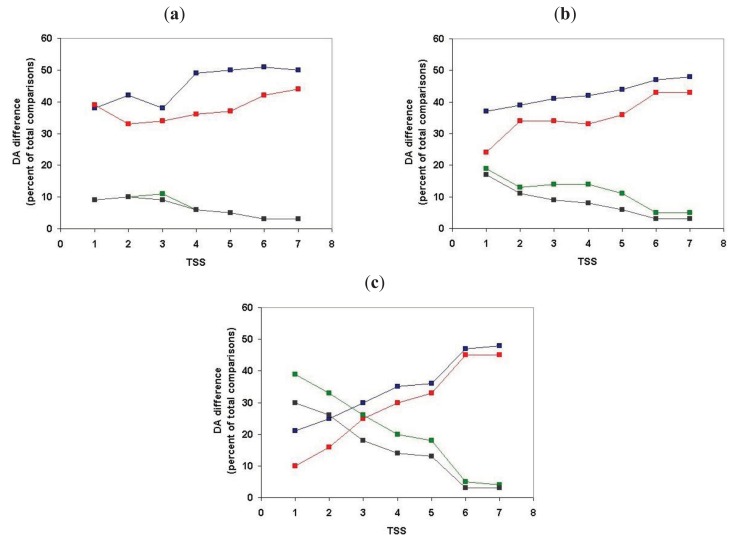
Analysis of the positional distribution of DA differences of paralogous human Swiss-Prot sequences differing in single domains. The numbers on the abscissa indicate the number of top-scoring matches included in the analyses (TSS = 1, …. TSS = 7) used to define paralogous clusters. N-terminal differences (blue recrangles), C-terminal differences (red rectangles), internal differences (green rectangles), tandem duplications (black rectangles). (a) Positional distribution of DA differences for type 1 transitions; (b) Positional distribution of DA differences for type 2 transitions (note that, in the case of the closest paralogs (TSS = 1), the proportion of terminal and internal DA differences are comparable); and (c) Positional distribution of DA differences for type 3 transitions. Note that in the case of closest paralogs (TSS = 1 and TSS = 2) the proportion of internal DA difference exceeds those of N-terminal or C-terminal changes.

Analysis of the positional distribution of DA differences in this dataset (Table S6) revealed that, in the case of type 1, type 2 and type 3 transitions, there is a slight but consistent preference for N-terminal over C-terminal DA change (see Figure 10a–c). This probably reflects the fact that even Swiss-Prot is contaminated with N-terminally truncated proteins.

Analysis of type 2 transitions (Figure 10b) has shown that, in the case of close paralogs (TSS = 1), C-terminal and internal DA changes are observed with similar frequency, suggesting that the probability of DA change is similar for terminal and internal positions.

Consistent with this interpretation, in the case of type 3 transitions (where there are more internal than N-terminal or C-terminal positions for DA change), there is a significant shift in favor of internal DA changes: in the case of TSS = 1 and TSS = 2 the proportion of internal DA changes exceeded those of the N-terminal or C-terminal changes (see Figure 10c).

Interestingly, with the inclusion of more and more distant paralogs the proportion of internal DA differences decreases and those of terminal changes increase continually. A possible explanation for this observation is that clusters containing more and more distant paralogs are increasingly contaminated with epaktologs. Since the DA of epaktologs is likely to differ in terminal positions this might explain the shift in favor of terminal DA differences. It seems unlikely, however, that such a contamination fully accounts for our observations. First, in these analyses we compared only the domain architectures of homologs that differed in single domains, thereby suppressing the impact of epaktologs. Second, the decrease is continuous and is observed in the case of TSS = 2, TSS = 3, TSS = 4, *i.e.*, clusters where contamination of clusters with epaktologs is not yet significant.

As an alternative explanation we may consider the possibility that, in the case of closer paralogs (*i.e.*, paralogs that arose as a result of more recent gene duplication), the probability of terminal and internal DA changes are similar, whereas in the case of distant paralogs (*i.e.*, paralogs that arose as a result of more ancient gene duplications) the probability of terminal DA changes may exceed those at internal positions. In terms of evolutionary mechanisms, this explanation would imply that, in the case of closer paralogs, domain-shuffling played a greater role in DA evolution than in the case of more distant paralogs where the contribution of fusion-type events could be more significant.

According to this interpretation, since the majority of close human paralogs was formed in the intron-rich genomes of early chordates exon-shuffling played a major role in shaping their DA [27] and since this evolutionary mechanism has a similar chance to add domains at internal or terminal positions, their DA evolution does not show a strong positional bias. Conversely, the fact that terminal DA changes dominate in the case of more distant human paralogs (that have formed prior to the divergence of chordates from other Metazoa) might reflect the fact that exon-shuffling has played a less significant role in the case of intron-poor genomes.

#### 2.2.1. Comparison of the DA of Paralogous Human Swiss-Prot Proteins Defined through Comparison of Refseq Sequences of Target Proteomes

**Figure 11:**
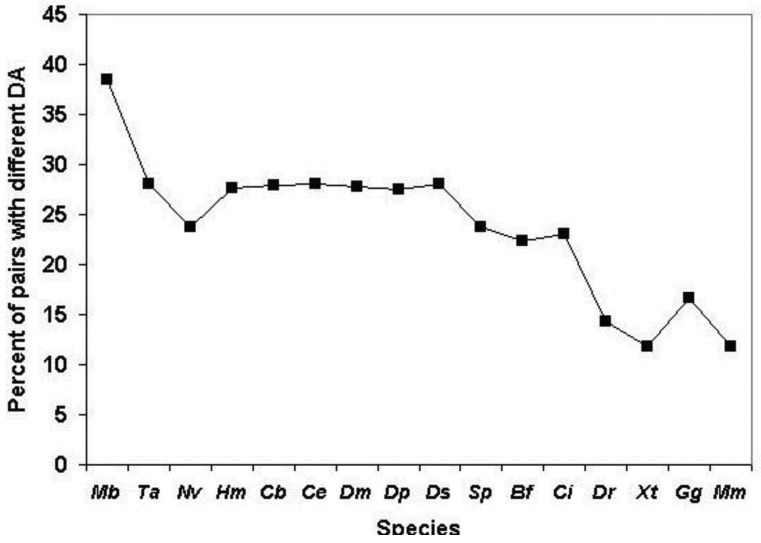
Analysis of the DA of paralogous clusters of human Swiss-Prot proteins defined through comparison with RefSeq proteomes of various species. The ordinate shows the proportion of pair-wise comparisons within clusters where the pairs differ in DA. On the abscissa the species are listed in the order of decreasing evolutionary distance from *Homo sapiens*, thus the abscissa has a time-dimension but their distance is not drawn to scale. Note that there is a significant drop in the proportion of pairs that differ in DA at the boundary of the invertebrate/vertebrate transition. Abbreviations on the abscissa: *Ta - Trichoplax adhaerens, Nv - Nematostella vectensis, Hm - Hydra magnipapillata, Ce -Caenorhabditis elegans, Cb -Caenorhabditis briggsae, Dm -Drosophila melanogaster, Dp -Drosophila pseudoobscura, Ds - Drosophila simulans, Sp - Strongylocentrotus purpuratus, Bf - Branchiostoma floridae, Ci - Ciona intestinalis, Dr - Danio rerio, Xt - Xenopus tropicalis, Gg - Gallus gallus, Mm - Mus musculus*.

To gain further insight into DA evolution of younger and older paralogs we have also characterized homologous clusters defined by the different target proteomes with respect to the heterogeneity or homogeneity of the DA of constituent paralogs. To estimate the degree of DA heterogeneity in clusters of paralogs we performed all-against-all comparisons within clusters and calculated the percent of comparisons that yielded DA differences (Table S7, Figure 11).

Analyses of the paralogous clusters of human Swiss-Prot proteins defined by various Placozoan, Cnidarian and Protostome proteomes have revealed that ∼28% of the comparisons yielded DA differences (Table S7 and Figure 11). The fact that the heterogenity of these clusters is very similar is consistent with the notion that these Metazoa define the same paralogous clusters: the ones that arose by duplications in the Deuterostome lineage (<910 Mya). In the case of the paralogous clusters defined by invertebrate deuterostomes (*Strongylocentrotus, Branchiostoma, Ciona*), the degree of DA heteogeneity of clusters is lower (∼23% of the cases), suggesting that some of the gene duplications giving rise to human paralogs occurred in early deuterostomes prior to the divergence of Echinoderms and Chordates (∼800 Mya). In the case of the (fewer) paralogous clusters defined by *Danio rerio, Xenopus tropicalis* and *Mus musculus* proteomes (clusters that arose after the fish/tetrapod split and the rodent/primate split), only 14%, 12% and 12% of the comparisons yielded DA differences, respectively. It should be noted that paralogous clusters defined by the incomplete proteome of *Gallus gallus* is slightly more heterogenous, probably reflecting the contamination of these clusters with more distant paralogs.

Since human paralogs defined by Placozoan, Cnidarian and Protostome proteomes arose by gene duplications <910 Mya, we can estimate % DA change/My to be (28%/<910) >0.031% DA change/My. Similarly, since human paralogs defined by invertebrate deuterostome proteomes arose by gene duplications <800 Mya, we can estimate % DA change/My to be (23%/<800) >0.029% DA change/My. It should be noted that these estimated rates of DA alteration are an order of magnitude higher than those calculated for orthologs (<0.005% DA change/My, see accompanying paper [4].

**Figure 12:**
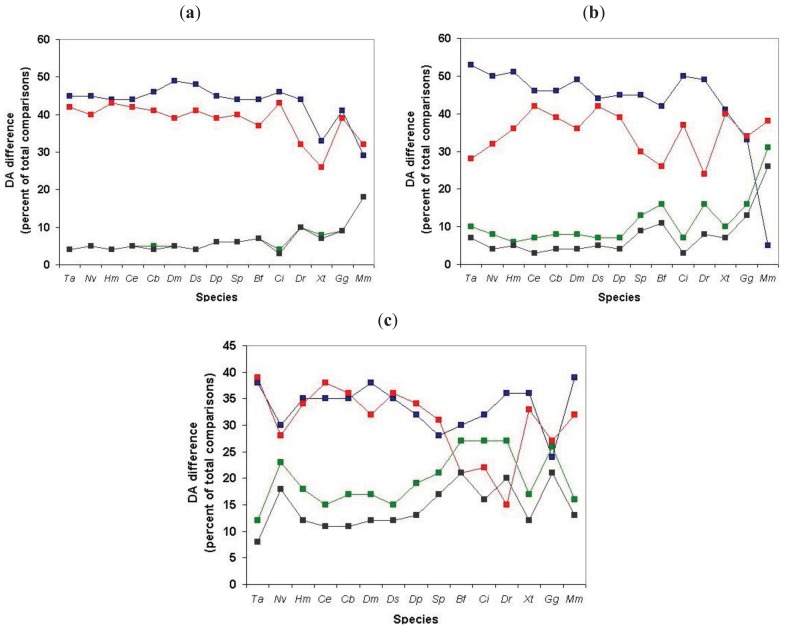
Analysis of the positional distribution of DA differences in clusters of human Swiss-Prot proteins defined through comparison with RefSeq proteomes of various species. The ordinate shows the proportion of pair-wise comparisons within clusters where the pairs differ in DA. N-terminal differences (blue rectangles), C-terminal differences (red rectangles), internal differences (green rectangles), tandem duplications (black rectangles). (a) Positional distribution of DA differences for type 1 transitions; (b) Positional distribution of DA differences for type 2 transitions; and (c) Positional distribution of DA differences for type 3 transitions. Note that in the case of type 3 transitions the proportion of internal DA difference is comparable to those of N-terminal or C-terminal changes only in the case of chordate species. On the abscissa the species are listed in the order of decreasing evolutionary distance from *Homo sapiens*, thus the abscissa has a time-dimension but their distance is not drawn to scale. Abbreviations on the abscissa: *Ta - Trichoplax adhaerens, Nv - Nematostella vectensis, Hm - Hydra magnipapillata, Ce -Caenorhabditis elegans, Cb -Caenorhabditis briggsae, Dm -Drosophila melanogaster, Dp -Drosophila pseudoobscura, Ds - Drosophila simulans, Sp - Strongylocentrotus purpuratus, Bf - Branchiostoma floridae, Ci - Ciona intestinalis, Dr - Danio rerio, Xt - Xenopus tropicalis, Gg - Gallus gallus, Mm - Mus musculus*.

When we analyzed the positional distribution of DA changes for type 1, type 2 and type 3 transitions within the paralogous clusters defined by the proteomes of the various species we noted a slight preference for N-terminal over C-terminal DA differences, irrespective of the species used to cluster paralogs (Table S8; Figure 12a and Figure 12b).

Interestingly, in the case of type 2 and type 3 transitions (Table S8, Figure 12b and Figure 12c) the proportion of internal DA change showed a strong dependence on the species used to cluster paralogs. In the case of type 2 transitions and clusters defined by various vertebrate proteomes (see chick-, zebrafish-defined clusters) and invertebrate deuterostome Refseq sequences (see *Branchiostoma*-, *Strongylocentrotus*-defined clusters), DA change of the internal type was significant (13–16% DA change), but these values were lower (∼7%) in the case clusters defined by RefSeq sequences of Protostome, Cnidarian and Placozoa proteomes (Figure 12b). This tendency was more pronounced in the case of type 3 transitions (Figure 12c). In the case of clusters defined by B*ranchiostoma floridae, Ciona intestinalis* and *Danio rerio* RefSeq sequences, ∼27% of DA change was of the internal type (comparable to the proportion of terminal changes), whereas in the case of clusters defined by RefSeq sequences of Protostome, Cnidaria and Placozoa proteomes this value was only ∼18% (much lower than the proportion of N-terminal or C-terminal DA changes). To put it in another way: in the case of paralogs clustered by non-deuterosrome metazoa the contribution of internal DA changes is relatively low, whereas in the case of paralogs clustered by *Branchiostoma floridae, Ciona intestinalis* and *Danio rerio* RefSeq sequences the contributions of internal and terminal DA changes are very similar.

Taken at face value, the most plausible explanation for these changes in the relative contribution of internal DA changes is that the role of mechanisms that can insert domains in internal positions (e.g., exon-shuffling) became more significant in shaping the DA of human paralogs that arose in early chordates. The data are thus consistent with the view that a ‘burst’ in domain-shuffling played a major role in the creation of novel multidomain proteins unique to vertebrates [27].

## 3. Experimental

### 3.1. Databases

UniProtKB Swiss-Prot entries [28] were downloaded from [29]. Protein sequences were retrieved from the RefSeq database [30,31] database.

### 3.2. Analysis of the Structure of Sequence Similarity Networks

We analyzed the structure of sequence similarity networks of paralogs with the Pajek software version 1.26 [14-16].

### 3.3. Comparison of the Domain Architectures of Homologous Proteins

We used the protocol described in the accompanying paper [4]. Briefly, the domain architectures were determined by RPS-BLAST against the Conserved Domain Database using Pfam-derived position-specific scoring matrices. Domain hits with an e-value of <10^−5^ were recorded, overlapping hits were eliminated and the DA (linear sequence of domains with e value of <10^−5^) was determined. The DAs of homologous pairs were compared and in the case of DA difference their DA differences were recalculated using the programs of the HMMER 2.3.2 software package and the Pfam HMM libraries at four different e-value cut-offs: <10^−2^, <10^−3^, <10^−4^ and <10^−5^.

#### 3.3.1. Classification of Differences in Domain Architecture

As described in the accompanying paper, DA differences detected at four different cut-off values (e-value <10^−2^, <10^−3^, <10^−4^, <10^−5^) were classified with respect to number of Pfam A domains distinguishing DAs, number of Pfam A domain-types distinguishing DAs and the positions of Pfam A domains that distinguish the DAs relative to shared domain(s). In the latter case the pairs of homologs were assigned to the

(1) N-Terminal Domain Difference category;
(2) C-Terminal Domain Difference category;
(3) Internal Domain Difference category;
(4) Domain Duplication Difference category;
(5) Positionally Not Assigned category;
(6) Identical Domain Architecture category.

Note that, since our protocol of DA comparison uses four different cut-off values, four assignments are made for each homolog pair. In the most unambiguous cases of DA differences, the given pair is assigned four times to the same category but in many cases the pair may be assigned to different categories at different cut-off values. Also, note that a given pair may show more than one type of difference, therefore the given pair may be assigned to more than one category, therefore the sum-total of the assignments may be greater than 4-times the number of pairs compared.

We have also pointed out in the accompanying paper that the classification according to the positions of PfamA domains that distinguish the DAs may introduce a positional bias even if we assume that the probability of DA changes are similar at all positions of the multidomain protein outside the domain boundaries. In order to analyze the contribution of this factor to the positional distribution of DA changes, we have also categorized single domain DA changes whether they belong to the one-domain ↔ two domain transitions (type 1 transitions), the two-domain ↔ three domain transitions (type 2 transitions) and the N-domain ↔ N+1-domain transitions, where N is greater than 2 (type 3 transitions).

## 4. Conclusions

In this manuscript we have examined the impact of confusing paralogous and epaktologous multidomain proteins (*i.e.*, those that are related only through the independent acquisition of the same domain types) on conclusions drawn about DA evolution of multidomain proteins in Metazoa. We used two types of paralogy-group construction procedures and monitored the impact of various parameters on the separation of true paralogs from epaktologs on correctly annotated Swiss-Prot entries of multidomain proteins. In these studies we used UniProtKB/Swiss-Prot sequence families with well-characterized evolutionary histories.

Our studies have shown that analysis of the structure of sequence similarity networks of multidomain proteins provides an efficient means for the separation of epaktologs and paralogs. On the other hand, we have demonstrated that contamination of protein families with epaktologs significantly increases the apparent rate of DA change and distorts the results by introducing a strong positional bias in favor of terminal over internal DA changes.

Examination of representative cases suggested that the probability of confusing epaktologous and paralogous multidomain proteins is increased by the presence of tandem duplicated domains in the proteins compared. Several factors contribute to this correlation. First, in the case of small mobile domains (Kringle-, FN2-, TSP1-, Kazal-, SRCR-domains etc., average length <100 amino acids) the relatively low sequence similarity score per individual domain is significantly increased if these domains occur in tandem arrays (that may be aligned), thus epaktologs sharing arrays of small Pfam A domains may appear to be more closely related than paralogs (see Figure 2c and Figure 3). Second, in our previous work we have shown that there is an inverse relationship between the size of domains and the degrees of their promisciuty/versatility, *i.e.*, the majority of highly versatile domains tend to be small [2]. The preponderance of small domains among the domains that frequently participate in domain-shuffling thus provides an explanation for our observation that there is a correlation between tandem duplication of domains and epaktology. The fact that 17%–21% of human Swiss-Prot proteins contains at least two tandem copies of a Pfam A domain (using cut-off values of e-value <10^-5^ or e-value <10^-2^, respectively) indicates that the problem caused by epaktology may affect a large number of multidomain proteins.

Note that, in the case of larger mobile domains (e.g., OLF, average length 218 amino acids), the influence of domain-duplication is less significant. This explains why MYOC-HUMAN may be clustered with the epaktologous LPHN1_HUMAN, LPHN2_HUMAN and LPHN3_HUMAN via the solitary OLF domain present in these proteins (see section 2.1).

These findings caution that earlier studies based on analysis of datasets of protein families that were contaminated with epaktologs may have led to some erroneous conclusions about the evolution of novel domain architectures of multidomain proteins. A reassessment of the DA evolution of multidomain proteins is presented in an accompanying paper [1].

## Supplementary Materials

Figure S1 Comparison of the domain architectures of TPA_HUMAN and its closest human paralogs.

Figure S2 Comparison of the domain architectures of THRB_HUMAN and its closest human paralogs.

Figure S3 Comparison of the domain architectures of NETR_HUMAN and its closest human epaktologs.

Figure S4 Comparison of the domain architectures of TSP2_HUMAN and its closest human paralogs.

Figure S5 Comparison of the domain architecture of MYOC_HUMAN with those of its closests paralogs and some epaktologs.

Figure S6 Comparison of the domain architecture of MMP2_HUMAN with those of its closest paralogs and some epaktologs.

Figure S7 Comparison of the domain architecture of SE1L1_HUMAN with those of its closest paralogs and some epaktologs.

Figure S8 Comparison of the domain architecture of AGRIN_HUMAN with that of its closest paralog.

Figure S9 Comparison of the domain architecture of AGRIN_HUMAN with those of some of its epaktologs.

**Table S1:** Parameters of RefSeq datasets of various species and sequence similarity networks of Human Swiss-Prot/Refseq comparisons.

**Species**	**Gene**	**RefSeq**	**Coverage**	**Component**	**Vertex**
*Trichoplax adhaerens*	11514 [32]	11540	1.00	3628	14947
*Nematostella vectensis*	18000 [33]	24780	1.38	3771	13435
*Hydra magnipapillata*	17250 [34]	17398	1.01	3419	14332
*Caenorhabditis elegans*	20621 [35]	23906	1.16	3331	13559
*Caenorhabditis briggsae*	19507 [35]	19416	1.00	3266	14360
*Drosophila melanogaster*	14422 [36]	16688	1.16	3747	13766
Drosophila simulans	16003 [36]	15428	0.96	3585	15077
*Drosophila pseidoobscura*	16388 [36]	16071	0.98	3649	14880
*Strongylocentrotus_purpu ratus*	23300 [37]	42324	1.82	3671	13255
*Branchiostoma floridae*	21900 [38]	28639	1.31	3893	13055
*Ciona intestinalis*	16000 [39]	13065	0.82	4014	14748
*Danio rerio*	32.174 [40]	27845	0.87	3178	9034
*Xenopus tropicalis*	20.500 [40]	8564	0.42	4283	16136
*Gallus gallus*	23.212 [41]	18792	0.81	3964	11411
*Mus musculus*	22011 [42]	35989	1.64	1628	4419
*Homo sapiens*	20500 [43]	38292	1.87	193	419

Species - the RefSeq proteomes of these species were used to cluster paralogous human Swiss-Prot sequences. Gene - number of predicted genes in the given species (numbers in parenthesis refer to the references listed below). RefSeq - number of entries in the RefSeq proteome for the given species; Coverage - RefSeq/Gene, parameter reflecting the incompleteness or redundancy of the RefSeq proteome of the given species. (Note that since a dataset may be redundant and incomplete at the same time, a coverage value close to 1.0 does not guarantee that the dataset is complete and non-redundant). Component - number of components in the sequence similarity network of the comparison of the RefSeq proteome of the given species with human Swiss-Prot entries; Vertex: number of human Swiss-Prot entries in the network that are connected by sequences of the target genome.

**Table S2:** Comparison of the DA of paralogous human Swiss-Prot proteins defined through comparison of human Swiss-Prot sequences.

**Dataset of Human Paralogs***	**Number of Paralogous Pairs Compared****	**Number of Pairs with Different DA**	**Percent of Pairs with Different DA**
TSS=1	4660	360	7,7
TSS=2	9033	1049	11,6
TSS=3	16764	2097	12,5
TSS=4	26592	6226	23,4
TSS=5	38074	10871	28,55
TSS=6	89402	51200	57,27
TSS=7	118857	71744	60.36

* Datasets containing the top-scoring one, two… seven sequences (TSS=1, TSS=2….TSS=7 datasets) were created as described in the text.

** Since the number of pair-wise com parisons and computational time increased exponentially with the increase of TSS values, the table shows only data for TSS=1….TSS=7.

**Table S3:** Positional distribution of DA differences of paralogous human Swiss-Prot proteins defined through intraspecies comparisons.

**Type of DA Difference****						

**Dataset***	**Nterm**	**Cterm**	**Internal**	**Duplication**	**NA**	**Identical**
TSS=1	37,7	29,1	3,7	21,4	3,6	4,6
TSS=2	43,3	31,0	2,7	16,4	3,6	3,0
TSS=3	41,7	37,3	3,4	11,1	5,0	1,5
TSS=4	45,0	38,4	2,8	8,3	4,7	0,8
TSS=5	46,1	40,9	2,1	6,3	4,0	0,6
TSS=6	48,5	46,7	0,5	1,5	2,6	0,1
TSS=7	48,5	46,6	0,5	1,3	3,0	0,1

* Datasets containing the top-scoring one, two… seven sequences (TSS=1, TSS=2….TSS=7 datasets) were created as described in the text.

** The numbers in the different categories represent the percen t of total assignments

**Table S4:** Proportion of homologous pairs of human Swiss-Prot sequences that differ in 1, 2, 3, or ≥4 domains.

**Number of Domains Distinguishing DAs of Homologous Pairs****				

**Dataset***	**N=1**	**N=2**	**N=3**	**N≥4**
TSS=1	66,50	15,09	7,54	10,86
TSS=2	56,98	19,97	7,73	15,32
TSS=3	51,80	20,26	8,68	19,27
TSS=4	45,67	18,51	9,35	26,47
TSS=5	41,82	18,68	9,49	30,00
TSS=6	20,76	13,43	9,64	56,17
TSS=7	21,48	14,40	9,90	54,23

* Datasets of paralogous human Swiss-Prot proteins containing the top-scoring one, two… seven sequences (TSS=1, TSS=2….TSS=7 datasets) were created as described in the text.

** The numbers in the different categories represent the percent of total DA differences.

**Table S5:** Proportion of type 1, type 2 and type 3 transitions in the case of homologous pairs of human Swiss-Prot sequences that differ in one domain.

**Transition Type****			

**Dataset***	**Type 1**	**Type 2**	**Type 3**
TSS=1	45	23	32
TSS=2	47	25	28
TSS=3	49	25	26
TTS=4	49	26	25
TTS=5	50	26	25
TSS=6	44	21	35
TSS=7	43	22	34

* Datasets of paralogous human Swiss-Prot proteins containing the top-scoring one, two… seven sequences (TSS=1, TSS=2….TSS=7 datasets) were created as described in the text.

** Type 1 transition: one-domain ↔ two domain transition; type 2 transition: two-domain ↔ three domain transition; type 3 transitions: N-domain ↔ N+1-domain transitions, where N is greater than 3. The numbers in the different categories represent the percent of total DA differences.

**Table S6:** Positional distribution of DA differences of paralogous human Swiss-Prot proteins defined through intraspecies comparisons.

**Type of DA Difference****							
**Dataset***	**Transition Type**	**Nterm**	**Cterm**	**Internal**	**Duplication**	**NA**	**Identical**
TSS=1	1	38	39	9	9	5	0
	2	37	24	19	17	2	0
	3	21	10	39	30	1	0
TSS=2	1	42	33	10	10	5	0
	2	39	34	13	11	2	0
	3	25	16	33	26	1	0
TSS=3	1	38	34	11	9	8	0
	2	41	34	14	9	3	0
	3	30	25	26	18	1	0
TSS=4	1	49	36	6	6	3	0
	2	42	33	14	8	3	0
	3	35	30	20	14	1	0
TSS=5	1	50	37	5	5	3	0
	2	44	36	11	6	3	0
	3	36	33	18	13	1	0
TSS=6	1	51	42	3	3	2	0
	2	47	43	5	3	2	0
	3	47	45	5	3	1	0
TSS=7	1	50	44	3	3	1	0
	2	48	43	5	3	2	0
	3	48	45	4	3	1	0

* Datasets containing the top-scoring one, two… seven sequences (TSS=1, TSS=2….TSS=7 datasets) were created as described in the text.

** The numbers in the different categories represent the percen t of total assignments

**Table S7:** Comparison of the DA of paralogous human Swiss-Prot proteins defined through comparison with RefSeq proteomes of various species.

**Species***	**Number of Paralogous Pairs Compared**	**Number of Pairs with Different DA**	**Percent of Pairs with Different DA**
*Trichoplax adhaerens*	17615	4945	28,07
*Nematostella vectensis*	15706	3730	23,75
*Hydra magnipapillata*	17540	4836	27,57
*Caenorhabditis briggsae*	18087	5052	27,93
*Caenorhabditis elegans*	17526	4912	28,03
*Drosophila melanogaster*	17301	4795	27,72
*Drosophila pseudoobscura*	19596	5392	27,52
*Drosophila simulans*	19764	5542	28,04
*Strongylocentrotus purpuratus*	15646	3706	23,69
*Branchiostoma floridae*	15346	3423	22,31
*Ciona intestinalis*	17740	4083	23,02
*Danio rerio*	7347	1052	14,32
*Xenopus tropicalis*	3841	452	11,77
*Gallus gallus*	9864	1643	16,66
*Mus musculus*	3841	452	11,77

* The RefSeq proteomes of these species were used to defined clusters of paralogs as described in the text.

**Table S8:** Positional distribution of DA differences of paralogous human Swiss-Prot proteins defined through comparison with RefSeq proteomes of various species.

**Type of DA Difference****					
**Species***	**Transition Type**	**Nterm**	**Cterm**	**Internal**	**Duplication**
*Trichoplax adhaerens*	1	45	42	4	4
	2	53	28	10	7
	3	38	39	12	8
*Nematostella vectensis*	1	45	40	5	5
	2	50	32	8	4
	3	30	28	23	18
*Hydra magnipapillata*	1	44	43	4	4
	2	51	36	6	5
	3	35	34	18	12
*Caenorhabditis elegans*	1	44	42	5	5
	2	46	42	7	3
	3	35	38	15	11
*Caenorhabditis briggsae*	1	46	41	5	4
	2	46	39	8	4
	3	35	36	17	11
*Drosophila melanogaster*	1	49	39	5	5
	2	49	36	8	4
	3	38	32	17	12
*Drosophila simulans*	1	48	41	4	4
	2	44	42	7	5
	3	35	36	15	12
*Drosophila pseidoobscura*	1	45	39	6	6
	2	45	39	7	4
	3	32	34	19	13
*Strongylocentrotus purpuratus*	1	44	40	6	6
	2	45	30	13	9
	3	28	31	21	17
*Branchiostoma floridae*	1	44	37	7	7
	2	42	26	16	11
	3	30	21	27	21
*Ciona intestinalis*	1	46	43	4	3
	2	50	37	7	3
	3	32	22	27	16
*Danio rerio*	1	44	32	10	10
	2	49	24	16	8
	3	36	15	27	20
*Xenopus tropicalis*	1	33	26	8	7
	2	41	40	10	7
	3	36	33	17	12
*Gallus gallus*	1	41	39	9	9
	2	33	34	16	13
	3	24	27	26	21
*Mus musculus*	1	29	32	18	18
	2	5	38	31	26
	3	39	32	16	13

* The RefSeq proteomes of these species were used to defined clusters of paralogs as described in the text.

** The numbers in the different categories represent the percent of total assignments
