# Supplementary figures and images for: Reassessing Domain Architecture Evolution of Metazoan Proteins: Major Impact of Errors Caused by Confusing Paralogs and Epaktologs
Source: Genes (Basel). 2011 Aug 2;2(3):516–61. doi: 10.3390/genes2030516 (PMC3927612; doi:10.3390/genes2030516)

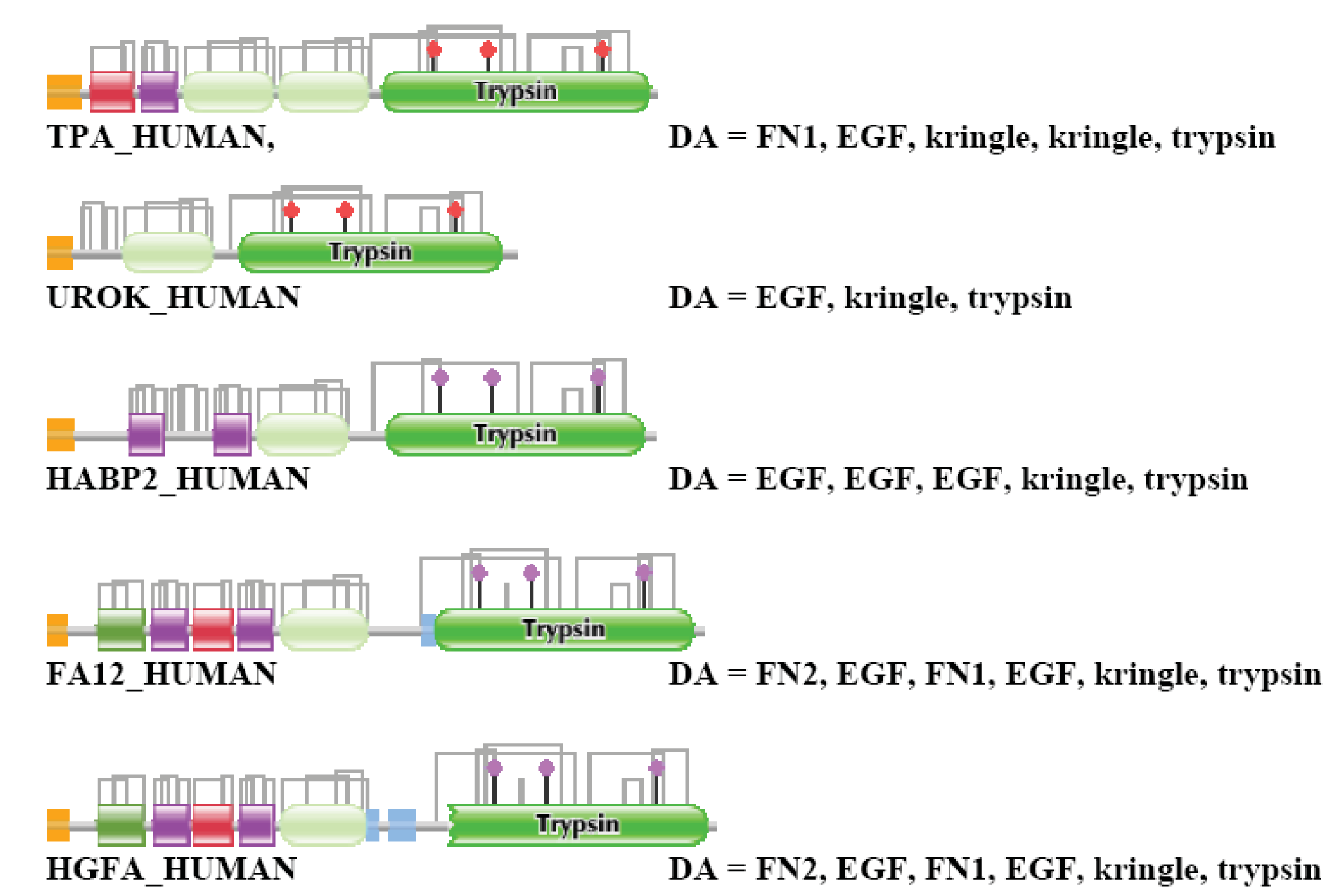

Supplement: Figure S1 — Comparison of the domain architectures of TPA_HUMAN and its closest human paralogs. [file genes-02-00516f13.tif]

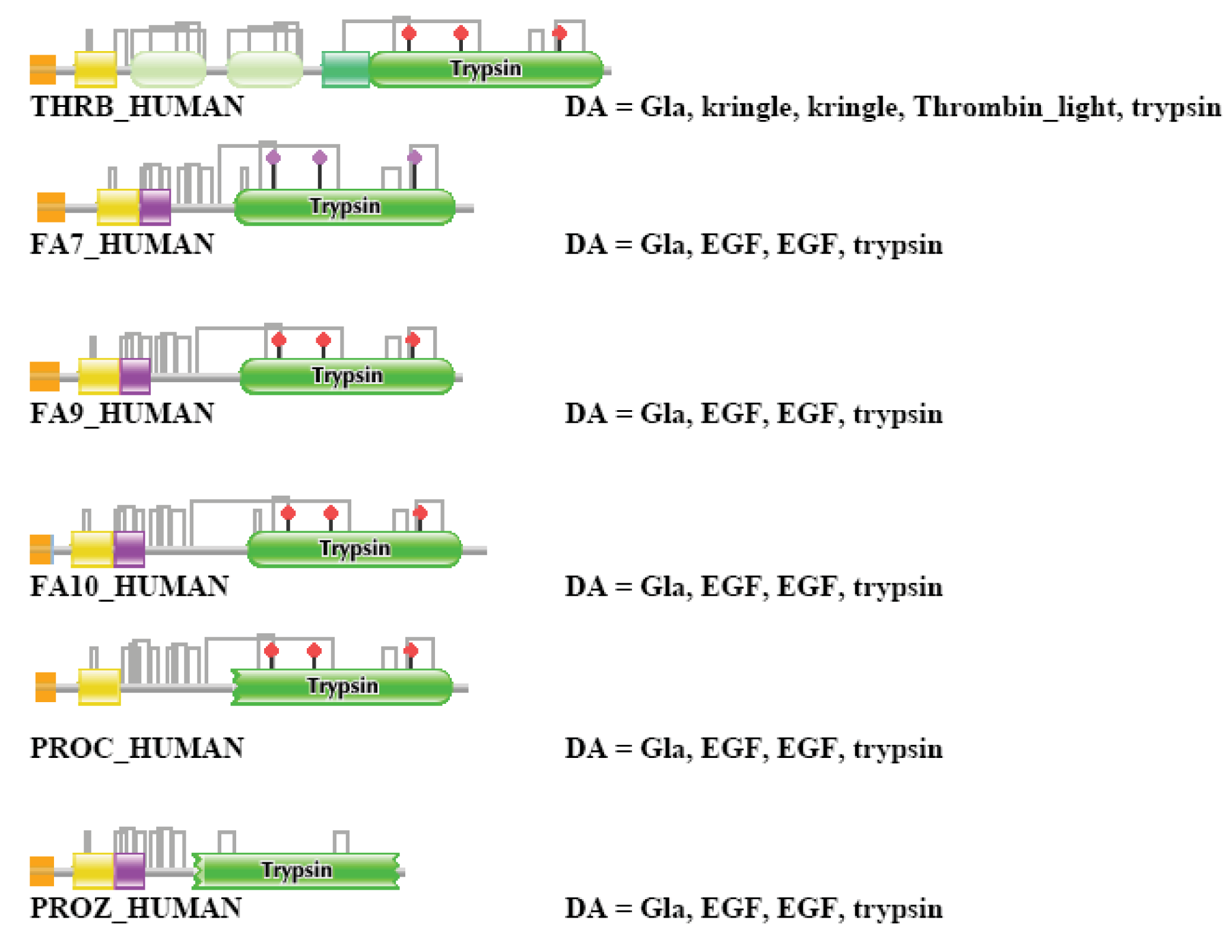

Supplement: Figure S2 — Comparison of the domain architectures of THRB_HUMAN and its closest human paralogs. [file genes-02-00516f14.tif]

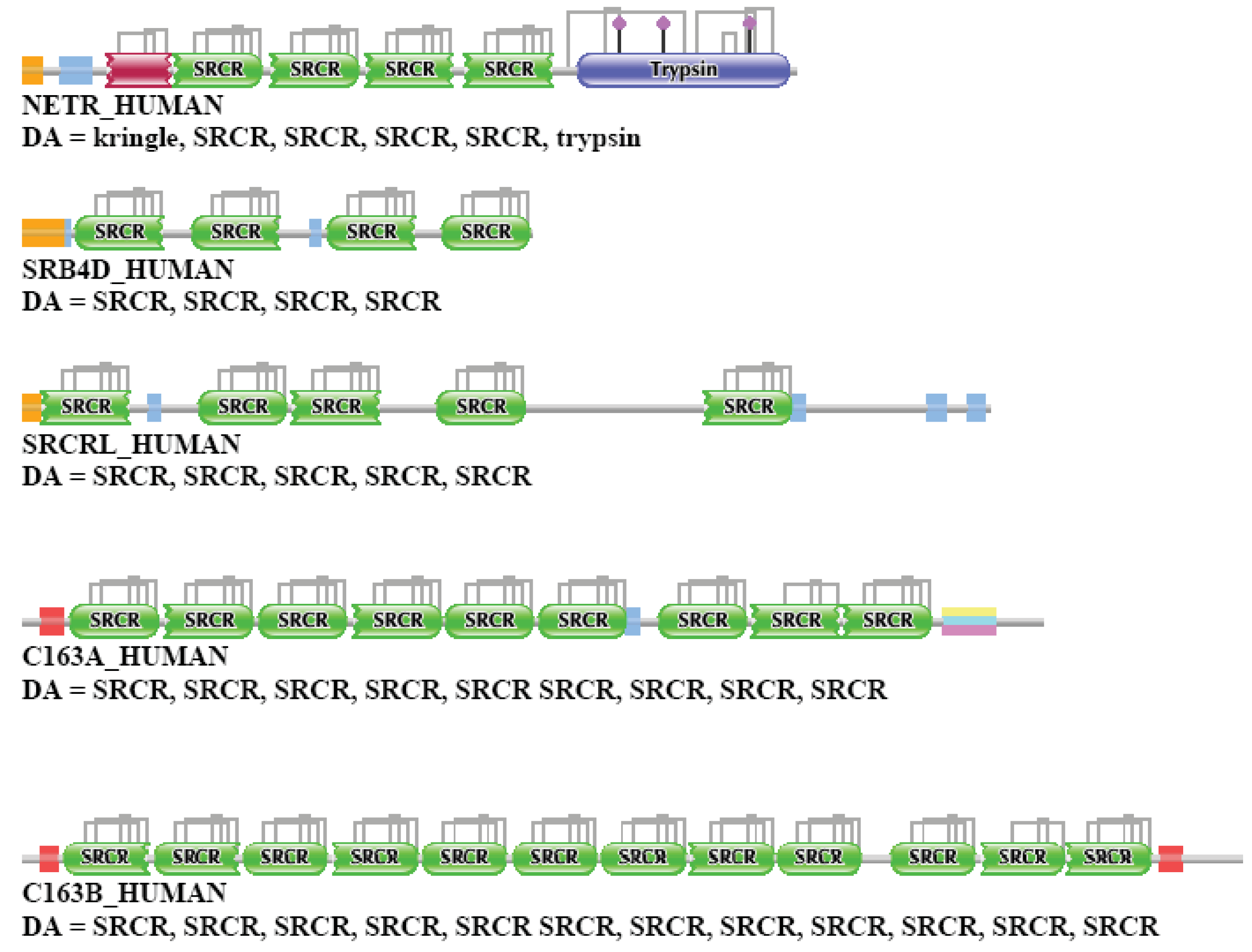

Supplement: Figure S3 — Comparison of the domain architectures of NETR_HUMAN and its closest human epaktologs. [file genes-02-00516f15.tif]

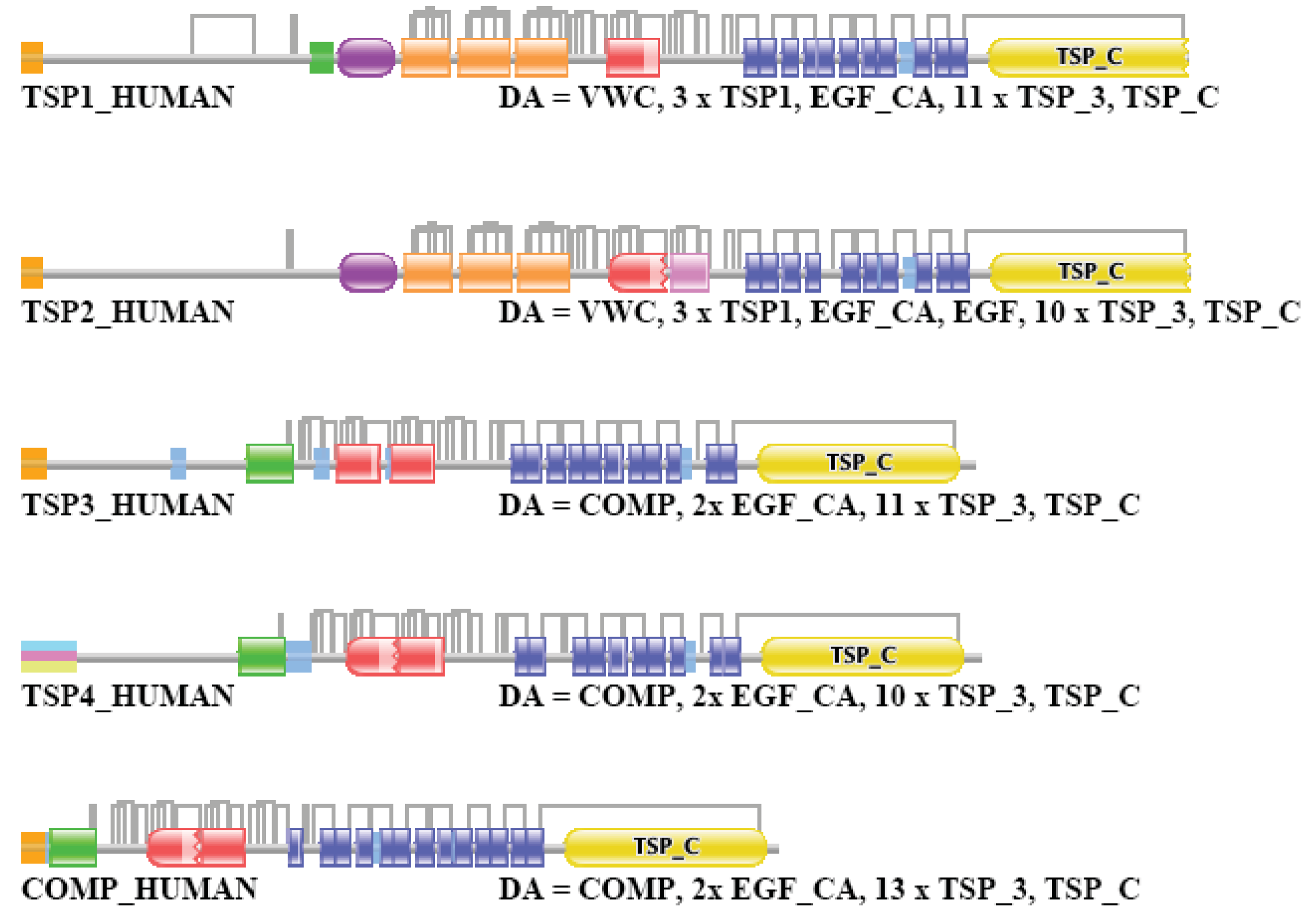

Supplement: Figure S4 — Comparison of the domain architectures of TSP2_HUMAN and its closest human paralogs. [file genes-02-00516f16.tif]

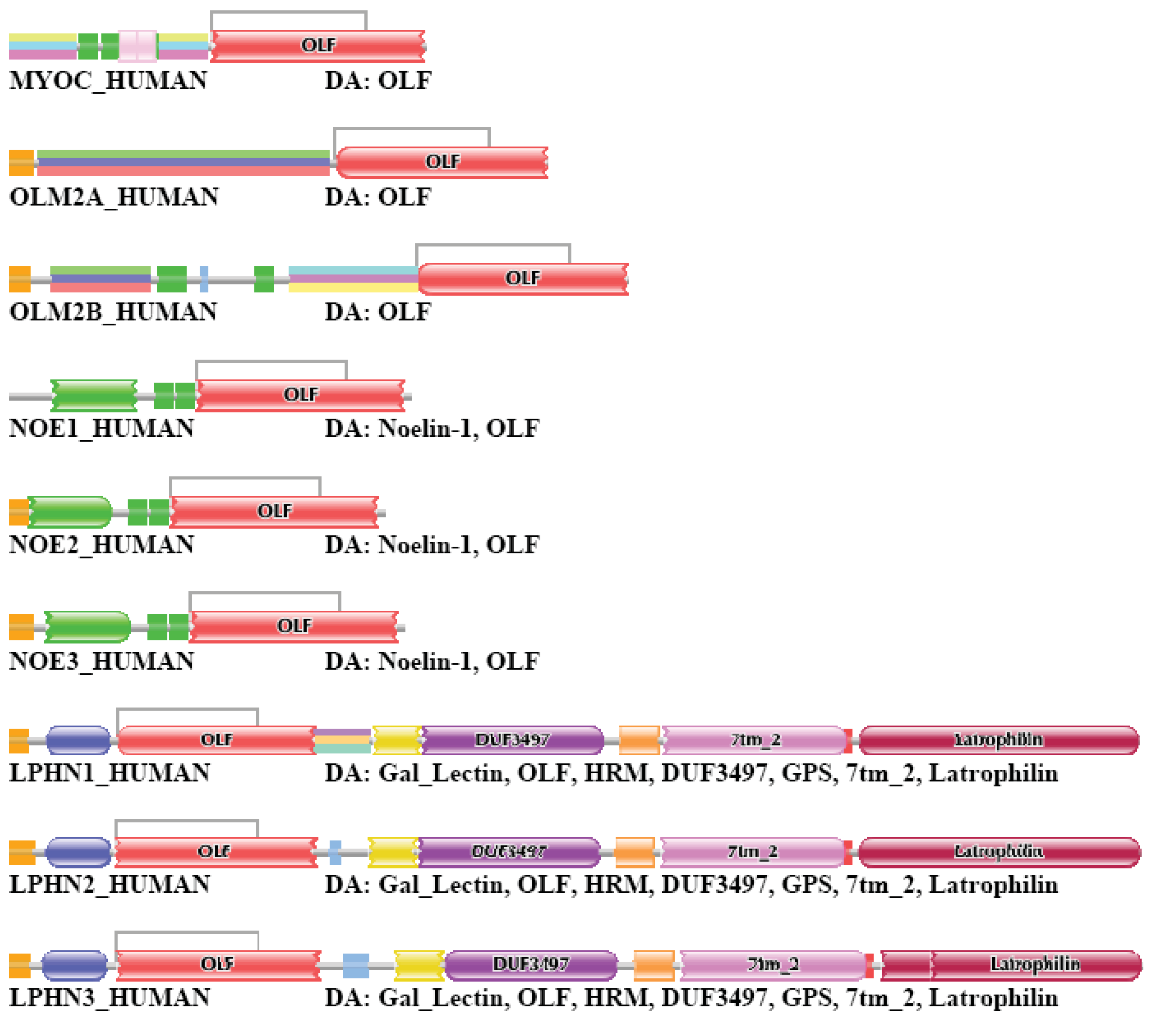

Supplement: Figure S5 — Comparison of the domain architecture of MYOC_HUMAN with those of its closests paralogs and some epaktologs. [file genes-02-00516f17.tif]

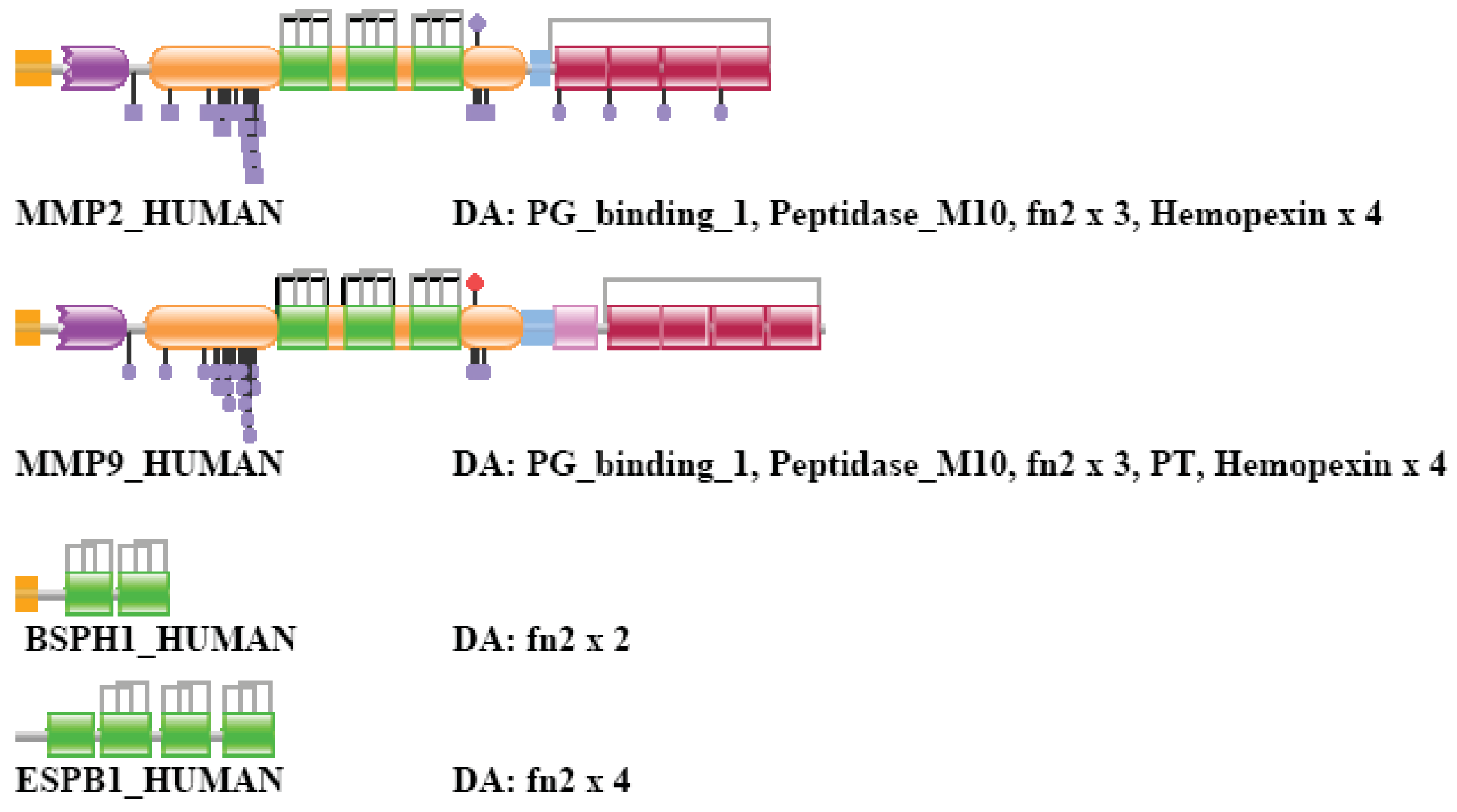

Supplement: Figure S6 — Comparison of the domain architecture of MMP2_HUMAN with those of its closest paralogs and some epaktologs. [file genes-02-00516f18.tif]

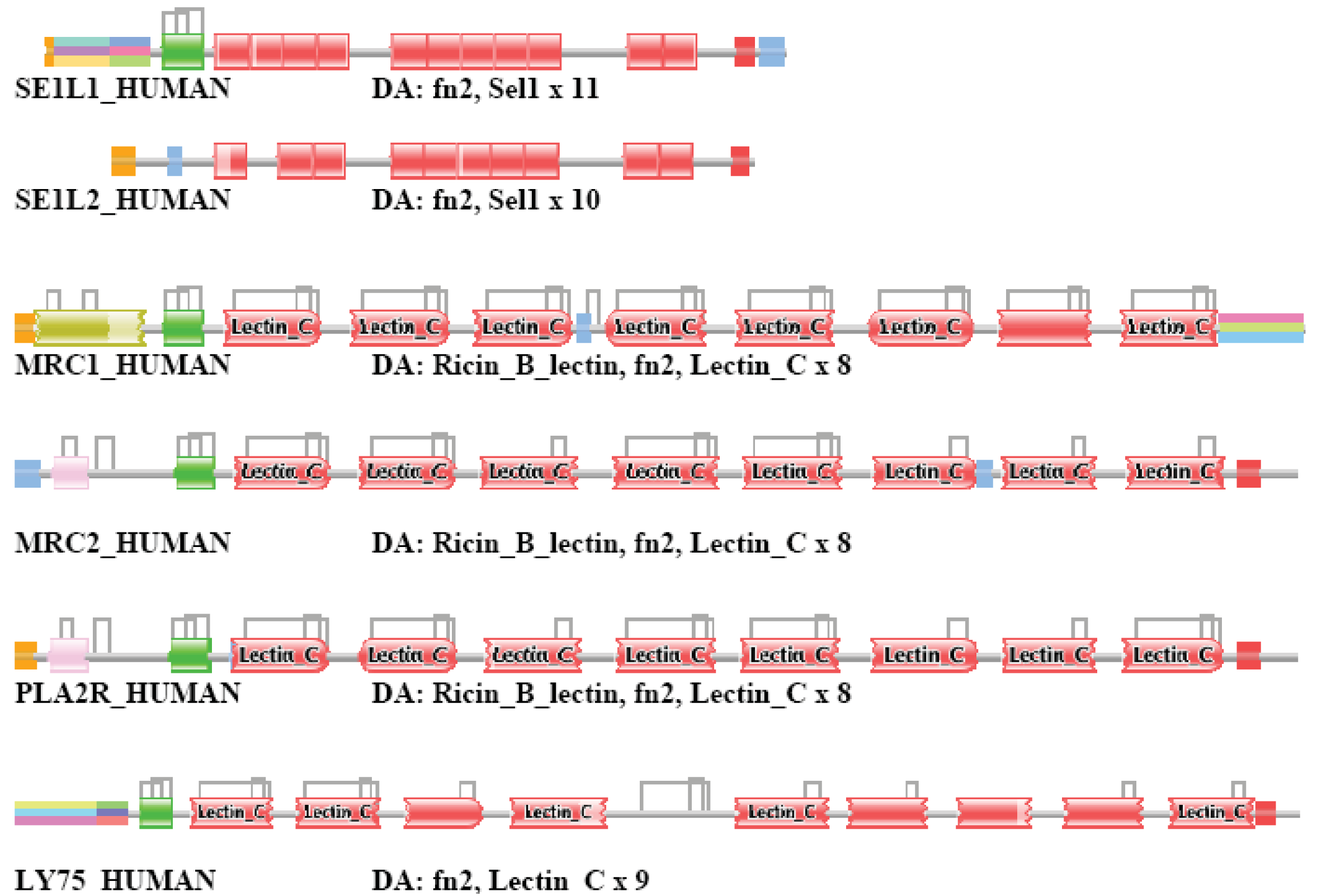

Supplement: Figure S7 — Comparison of the domain architecture of SE1L1_HUMAN with those of its closest paralogs and some epaktologs. [file genes-02-00516f19.tif]

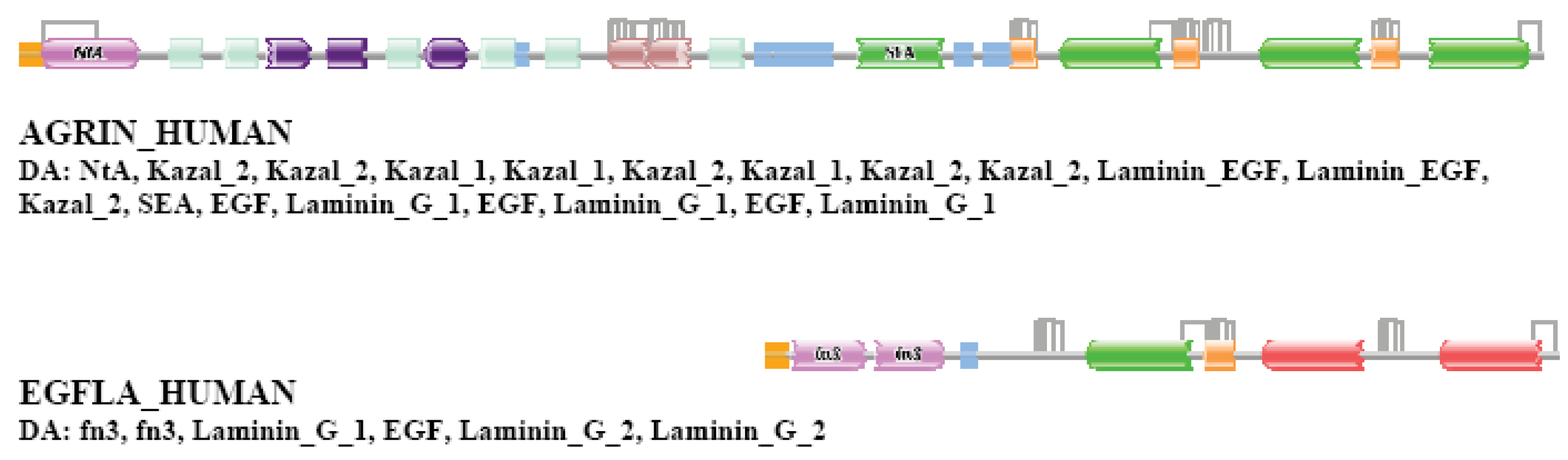

Supplement: Figure S8 — Comparison of the domain architecture of AGRIN_HUMAN with that of its closest paralog. [file genes-02-00516f20.tif]

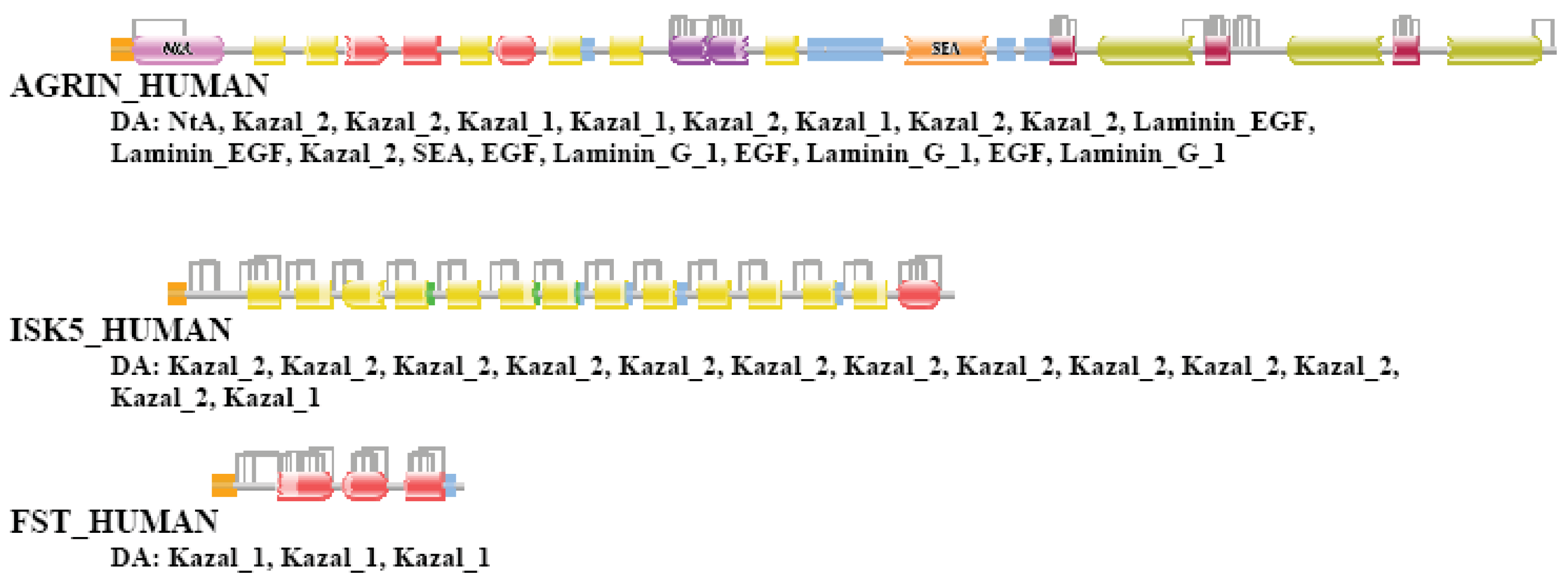

Supplement: Figure S9 — Comparison of the domain architecture of AGRIN_HUMAN with those of some of its epaktologs. [file genes-02-00516f21.tif]
